# Synchronous Disintegration of Ferroptosis Defense Axis via Engineered Exosome‐Conjugated Magnetic Nanoparticles for Glioblastoma Therapy

**DOI:** 10.1002/advs.202105451

**Published:** 2022-05-04

**Authors:** Boyan Li, Xin Chen, Wei Qiu, Rongrong Zhao, Jiazhi Duan, Shouji Zhang, Ziwen Pan, Shulin Zhao, Qindong Guo, Yanhua Qi, Wenhan Wang, Lin Deng, Shilei Ni, Yuanhua Sang, Hao Xue, Hong Liu, Gang Li

**Affiliations:** ^1^ Department of Neurosurgery Qilu Hospital Cheeloo College of Medicine and Institute of Brain and Brain‐Inspired Science Shandong University Jinan 250012 P. R. China; ^2^ State Key Laboratory of Crystal Materials Shandong University Jinan 250100 P. R. China; ^3^ Institute for Advanced Interdisciplinary Research (IAIR) University of Jinan Jinan 250022 P. R. China

**Keywords:** blood–brain barrier, exosomes, ferroptosis, glioblastoma, magnetic nanoparticles

## Abstract

Glioblastoma (GBM) is one of the most fatal central nervous system tumors and lacks effective or sufficient therapies. Ferroptosis is a newly discovered method of programmed cell death and opens a new direction for GBM treatment. However, poor blood–brain barrier (BBB) penetration, reduced tumor targeting ability, and potential compensatory mechanisms hinder the effectiveness of ferroptosis agents during GBM treatment. Here, a novel composite therapeutic platform combining the magnetic targeting features and drug delivery properties of magnetic nanoparticles with the BBB penetration abilities and siRNA encapsulation properties of engineered exosomes for GBM therapy is presented. This platform can be enriched in the brain under local magnetic localization and angiopep‐2 peptide‐modified engineered exosomes can trigger transcytosis, allowing the particles to cross the BBB and target GBM cells by recognizing the LRP‐1 receptor. Synergistic ferroptosis therapy of GBM is achieved by the combined triple actions of the disintegration of dihydroorotate dehydrogenase and the glutathione peroxidase 4 ferroptosis defense axis with Fe_3_O_4_ nanoparticle‐mediated Fe^2+^ release. Thus, the present findings show that this system can serve as a promising platform for the treatment of glioblastoma.

## Graphical abstract

A novel composite therapeutic platform combining the magnetic targeting features and drug delivery properties of magnetic nanoparticles with the blood–brain barrier penetration abilities and siRNA encapsulation properties of engineered exosomes for Glioblastoma therapy is presented. Synergistic ferroptosis therapy is achieved by the combined actions of the disintegration of ferroptosis defense axis with Fe3O4 nanoparticle‐mediated Fe2+ release.

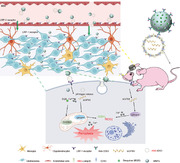

## 1 Introduction

Glioblastoma (GBM) is one of the most aggressive brain tumors, and has a poor prognosis.^[^ ^1^ ^]^ Currently, standard treatment measures for GBM include surgery followed by concurrent radiotherapy with temozolomide.^[^ ^2^ ^]^ Other treatment modalities, such as gene therapy,^[^ ^3^ ^]^ immunotherapy,^[^ ^4^ ^]^ and even tumor‐treating fields (TTFields),^[^ ^5^ ^]^ have not greatly extended the survival time of patients.

Ferroptosis is a newly discovered form of regulated cell death that is induced by excessive lipid peroxidation, and the mechanism of its occurrence and development is still being investigated.^[^ ^6^ ^]^ Glutathione peroxidase 4 (GPX4) is a key regulator of ferroptosis. In essence, ferroptosis involves the depletion of glutathione (GSH) and a decrease in the activity of GPX4, which does not allow lipid oxides to be metabolized by the GPX4‐catalyzed glutathione reductase reaction. Finally, divalent iron ions oxidize lipids to produce reactive oxygen species (ROS) and promote ferroptosis.^[^ ^7^ ^]^ Researchers have developed inhibitors against GPX4 and other ferroptosis‐inducing drugs, such as RSL3, ML210, and erastin.^[^ ^8^ ^]^ However, due to the existence of the blood–brain barrier (BBB) and inadequate tumor vascularization, it is difficult for ferroptosis drugs to reach the tumor and maintain a therapeutic dose.^[^ ^3^ ^]^ Most importantly, there is a compensatory mechanism against GPX4. Fortunately, the latest research published in *Nature* has revealed a new therapeutic target for ferroptosis, dihydroorotate dehydrogenase (DHODH).^[^ ^9^ ^]^ This new ferroptosis defense system works in parallel to GPX4 in mitochondria. After GPX4 inhibition, the level of DHODH is elevated to compensate for and counteract cell ferroptosis; in brief, the loss of one of these systems forces cells to rely on the other system (Figure S1, Supporting Information). Moreover, inhibition of DHODH induced ferroptosis in cells with low GPX4 expression was found; however, in cells with high GPX4 expression, DHODH inhibition did not significantly stimulate the onset of ferroptosis but instead caused the cells to become more sensitive to ferroptosis inducers.^[^ ^9^ ^]^ In contrast, simultaneous knockdown of GPX4 and the use of DHODH inhibitors induced stronger lipid peroxidation and ferroptosis. Based on these recent theories, we found that at the cell line level in glioblastoma, most GBM cell lines had high expression levels of DHODH and GPX4 compared with normal human astrocytes (NHA) in Figure S2 (Supporting Information). Combined with the Cancer Genome Atlas (TCGA) GBM sequencing results, DHODH was found to be highly expressed and GPX4 was slightly elevated in GBM (Figure S3, Supporting Information). Therefore, the simultaneous loss of these two protective systems is the most effective trigger of ferroptosis in GBM.^[^ ^9^, ^10^ ^]^

How to effectively penetrate the BBB to effectively deliver drugs or genes for GBM treatment has long perplexed researchers.^[^ ^11^ ^]^ Recent breakthroughs in nanotechnology have yielded versatile therapeutic nanoplatforms with the ability to cross the BBB, enabling precise diagnosis and effective treatment for GBM. For example, the first medical‐industrial crossover platform for GBM, the Gliadel wafer, was implanted in the tumor cavity for chemotherapy.^[^ ^12^ ^]^ However, this treatment system has the potential to induce cerebrospinal fluid secretion and intracranial infection. Subsequently, strategies based on focused ultrasound, microwaves, lasers, and electromagnetic fields have also been developed, but studies have shown that physical action‐based strategies to penetrate the BBB can disrupt its structure as the permeability is enhanced, leading to the penetration of harmful substances.^[^ ^13^ ^]^ Therefore, the ideal BBB penetration method should be controllable, reversible, and selective.

Exosomes are a class of extracellular vesicles (EVs) ≈30–150 nm in size that contain lipids, proteins, and nucleic acids and are used to respond to cellular origins and participate in intercellular communication,^[^ ^14^ ^]^ and exosomes considered to be an important vehicle for the treatment of many diseases, such as bone regeneration and tissue formation.^[^ ^15^ ^]^ In addition, exosomes have a natural biological advantage due to their biocompatibility and ability to penetrate the BBB which can be used in brain diseases.^[^ ^16^ ^]^ However, although exosomes show the ability to penetrate the BBB, many researchers believe that intravenously injected exosomes are mainly distributed in the liver or spleen, and a very small portion of injected exosomes are retained in the brain or in the tumor site. Therefore, a means for exosomes to target glioblastoma is needed. Physicochemical and genetic engineering methods can be used to modify exosomes to generate engineered exosomes for drug or gene delivery,^[^ ^17^ ^]^ such as by forming a fusion gene with Lamp2b protein for brain‐targeting for Parkinson's disease or ischemic stroke.^[^ ^18^ ^]^ However, RVG‐modified exosomes specifically target to neurons, microglia, and oligodendrocytes in the brain, but not glioblastoma cells. Angiopep‐2 (TFFYGGSRGKRNNFKTEEYC, ANG) is a peptide derived from the Kunitz domains of aprotinin that specifically binds low‐density lipoprotein receptor protein 1 (LRP‐1). LRP‐1 is highly expressed on brain capillary endothelial and glioma cells, and these characteristics make angiopep‐2 a promising candidate for LRP1‐mediated targeted drug delivery to glioblastoma.^[^ ^19^ ^]^ Therefore, we added ANG peptide to the fusion gene of Lamp2b to obtain ANG peptide‐modified engineered exosomes and obtain stronger BBB penetration and brain targeting ability. In addition, recent breakthroughs in nanotechnology have resulted in the creation of many nanoplatforms with the ability to cross the BBB.^[^ ^11^ ^]^ Many magnetic nanoparticles (MNPs), such as iron oxide nanoparticles (IONPs), can be used in drug delivery, magnetic resonance imaging, and tumor treatment.^[^ ^20^ ^]^ Therefore, we believe that the compounding of exosomes with magnetic nanoparticles can provide a better method for the targeted treatment of glioblastoma.

**Figure 1:**
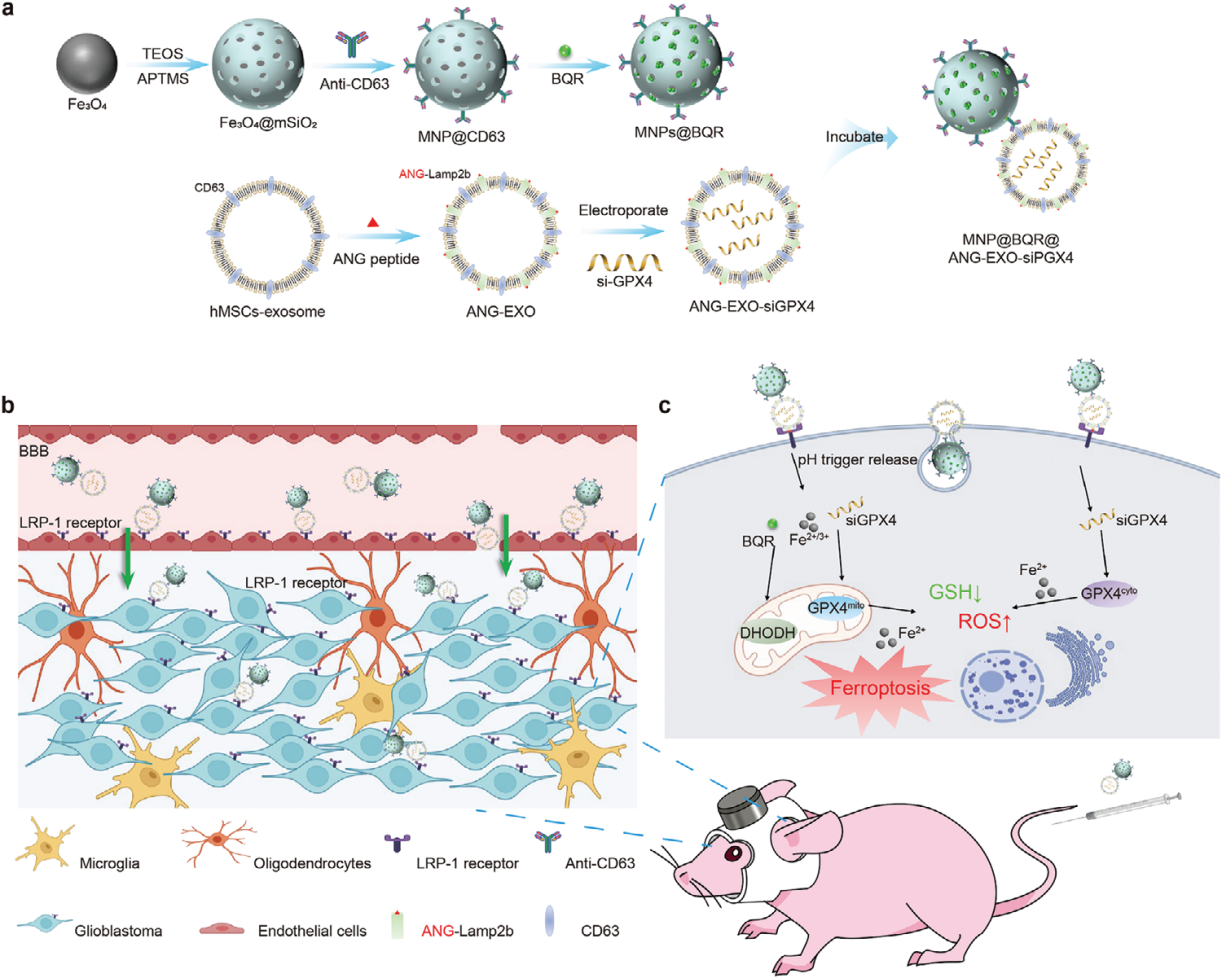
a) Schematic illustration of the design and synthesis of MNP@BQR@ANG‐EXO‐siGPX4. b) Schematic of the magnetic mouse helmet and the mechanisms by which the ANG peptide‐mediated NPs crossed the BBB to accumulate in tumors. c) The mechanism underlying the induction of GBM cell ferroptosis.

Herein, we designed a composite therapeutic platform combining the magnetic targeting features and drug delivery properties of MNPs with the BBB penetration abilities and small interfering RNA encapsulation properties of engineered exosomes for GBM therapy. This platform is composed of engineered exosome‐conjugated MNPs that can enhance ferroptosis by disintegrating the DHODH and GPX4 ferroptosis defense axes. The platform consists of an Fe_3_O_4_ core and a mesoporous silica shell that is conjugated with a CD63 antibody that binds to CD63 antigens on the surface of EVs. The original EVs were derived from human mesenchymal stem cells (hMSCs), and then an ANG peptide‐decorated exosome (ANG‐EXO) was produced by incorporation of ANG into the exosome membrane as an ANG and Lamp2b fusion protein. Small interfering RNA (siRNA) of GPX4 (siGPX4) was loaded into the exosomes by electroporation. Additionally, the surface of the mesoporous silica shell was incubated with brequinar (BQR), an inhibitor of DHODH that has been approved by the FDA and used in clinical practice (**Figure** **1**a). Magnetic helmets were constructed for nude mice using 3D printing techniques (Video S1, Supporting Information). After the application of a local magnetic field, the MNP@BQR@ANG‐EXO‐siGPX4 platform was first enriched in blood vessels in the brain followed by penetration of the BBB by recognition of the ANG targeting peptide by low‐density lipoprotein receptor‐related‐1 protein (LRP‐1) receptors. Moreover, this system recognizes GBM cells overexpressing LRP‐1 receptors,^[^ ^21^ ^]^ allowing the transport of these complexes (Figure 1b). Synergistic GBM treatment was achieved through the combined triple actions of disintegration DHODH and the GPX4 ferroptosis defense mechanism and Fe_3_O_4_ NPs‐mediated Fe^2+^ release (Figure 1c). In conclusion, this platform provides a new idea for enhanced ferroptosis for synergistic GBM therapy.

## 2 Results and Discussion

### 2.1 Preparation and Synthesis of Magnetic NPs

**Figure 2:**
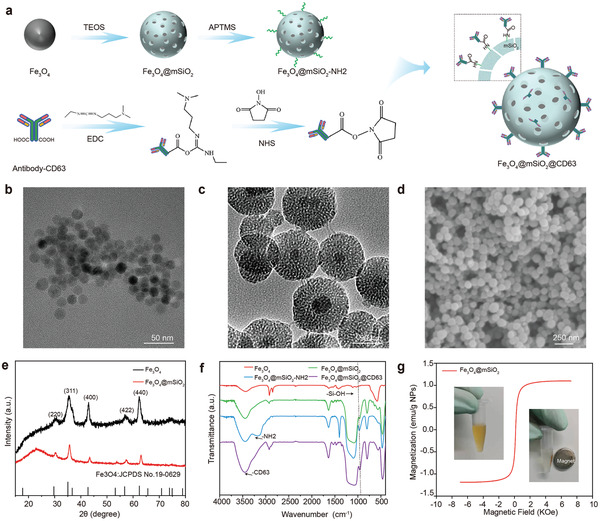
Synthesis of MNPs and their characterization. a) Schematic diagram of MNP preparation. The conjugation process between the amino‐functionalized MNPs and antibody‐CD63 was successful. b) Representative TEM images of the Fe_3_O_4_ NPs and c) MNPs. d) Representative SEM images of the MNPs. e) XRD analysis of the Fe_3_O_4_ NPs and Fe_3_O_4_@mSiO2. The JCPDS number is No.19‐0629. f) FTIR spectra of Fe_3_O_4_, and the Fe_3_O_4_ @mSiO_2_, Fe_3_O_4_ @mSiO_2_‐NH_2_‐, and Fe_3_O_4_ @mSiO_2_@CD63 NPs. g) Field‐dependent magnetization curve of Fe_3_O_4_ @mSiO_2_ at room temperature. Insets are images of the magnetic response of Fe_3_O_4_ @mSiO_2_.

To endow the DHODH inhibitor brequinar (BQR) magnetic targeting properties and perform exosomal delivery, antibody‐modified core–shell NPs consisting of Fe_3_O_4_ nanoparticles@mesoporous silica (Fe_3_O_4_@mSiO_2_ NPs, MNPs) were designed and prepared (**Figure** **2**a). Briefly, after coating the Fe_3_O_4_ NPs with mesoporous SiO_2_, the MNP surface was modified with (3‐aminopropyl) trimethoxysilane (APTMS). Additionally, the CD63 antibody (anti‐CD63) was modified with 1‐(3‐dimethylaminopropyl)‐3‐ethylcarbodiimide hydrochloride (EDC) and *N*‐hydroxysuccinimide (NHS). Then, the MNPs@anti‐CD63 NPs were constructed through the reaction of the amino groups on the MNP surface and carboxyl groups on anti‐CD63. The transmission electron microscopy (TEM) image showed that the core Fe_3_O_4_ structure had a size of 20 nm (Figure 2b) and the mesoporous SiO2 shell structure layer had a thickness of 30–40 nm (Figure 2c). The scanning electron microscopy (SEM) image confirmed the homogeneous spherical morphology of the MNPs (Figure 2d). The size distribution and zeta potential of the purified NPs were determined using NP tracking analysis (NTA) in ZetaView (Figures S4 and S5, Supporting Information). The X‐ray diffraction (XRD) patterns of Fe_3_O_4_ and Fe_3_O_4_@mSiO_2_ were assigned to magnetite (PDF#19‐0629), and the broad peak between 20° and 30° was assigned to the amorphous mesoporous SiO_2_ shell (Figure 2e). The Fourier transform infrared (FTIR) spectra of Fe_3_O_4_, Fe_3_O_4_@mSiO_2_, Fe_3_O_4_@mSiO_2_‐NH2, and Fe_3_O_4_@mSiO_2_@CD63 are shown in Figure 2f. Comparing the spectra of Fe_3_O_4_ and Fe_3_O_4_@mSiO_2_, the band observed at ≈955 cm^−1^ was attributed to the bending vibrations of Si—OH, and the peak at 800 cm^−1^ was attributed to the stretching vibration of Si—O bonds, which indicated the presence of mesoporous SiO_2_. After modification with APTMS, the double peaks in the 3000–3500 cm^−1^ region were assigned to the symmetric and antisymmetric stretching vibrations of —NH2. These results indicated the successful surface modification with —NH2 to form Fe_3_O_4_@mSiO_2_‐NH2. After reaction with modified anti‐CD63 (Fe_3_O_4_@mSiO_2_@CD63), the double peaks of —NH2 disappeared, and a strong single peak in the vicinity of 3500 cm^−1^ appeared. This peak was recognized as the amide bond that had formed between the amino group of the MNPs and the carboxyl group of anti‐CD63. The saturation magnetization curve of the MNPs showed closed hysteresis loops, demonstrating the superparamagnetic properties of the MNPs (Figure 2g). The saturation magnetization curve of Fe_3_O_4_ and Fe3O4@mSiO2 showed in Figure S7 (Supporting Information). Because of the lower mass ratio of Fe:Si (0.08:1), the amount of Fe3O4 in Fe3O4@mSiO2 is ≈5wt% and the content of Fe element in Fe3O4 and Fe3O4@mSiO2 is 72.4% and 3.6%. The saturation magnetization of the MNPs at room temperature was ≈1.2 emu g^−1^ NPs, which is sufficient to provide precise magnetic targeting properties for BQR and allow its exosomal delivery. We used T2‐weighted MRI to verify the magnetic properties of MNPs, as shown in Figure S8 (Supporting Information), due to the presence of Fe, the MNPs shown a concentration‐dependent signal reduction in T2‐weighted MRI. These results demonstrated the successful fabrication of MNPs.

### 2.2 Synthesis of ANG‐EXO

**Figure 3:**
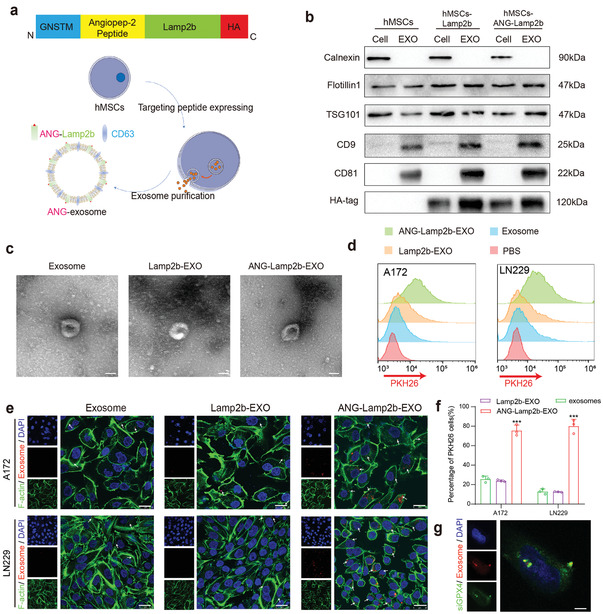
Synthetic process and biological functions of ANG‐EXO. a) Schematic diagram of DNA plasmid construction and the transfection process used to produce ANG‐EXO. b) Western blot analysis was performed on hMSCs, hMSCs‐Lamp2b, or hMSCs‐ANG‐Lamp2b and released exosomes. c) TEM image of the native exosomes, Lamp2b‐EXO and ANG‐Lamp2b‐EXO. d) Flow cytometry analysis of A172 and LN229 cells after incubation with PBS, exosome, Lamp2b‐EXO or ANG‐Lamp2b‐EXO. Exosomes were stained with PKH26 (red). e) Confocal microscopy of the cellular uptake of exosomes, Lamp2b‐EXO or ANG‐Lamp2b‐EXO after 6 h of incubation with A172 and LN229 cells. Staining is as follows: exosomes, PKH26 (red); F‐actin (cytoskeleton, green); and DAPI (nucleus, blue). Scale bar, 25 µm. f) Quantification of the PKH26‐positive cell ratio based on the confocal microscopy images (Data are presented as mean ± SD; *n* = 3; ***p < 0.001, compared with exosomes group). g) Confocal microscopy images of the electroporated ANG‐EXO‐siGPX4 cocultured with LN229 cells after 24 h. Exosome (red, PKH26) colocalization with siGPX4 (green, FAM) is highlighted. Scale bar, 5 µm.

EVs are cell membrane‐derived particles that have recently been extensively studied.^[^ ^15^, ^16^ ^]^ Viable proteins and nucleic acid substances carried in EVs play crucial roles in biological signaling. Most importantly, recent studies have demonstrated that exosome‐mediated cell delivery can be used as an effective treatment strategy.^[^ ^17a^ ^]^ Specific signaling molecules, nucleic acid substances, and drugs can be introduced into cell‐derived exosomes with certain modifications for the treatment of diseases. Exosomes have good biocompatibility and targeting properties, and exosome‐based nanocarriers are widely used in clinical applications. Researchers use chemical, cellular or genetic engineering techniques to modify exosomes for this purpose.^[^ ^22^ ^]^ In our article, we used the Lamp2b gene fusion approach for exosome modification. The method of peptide fusion with Lamp2b was first proposed in 2011 to delivery siRNA to the brain,^[^ ^18a^ ^]^ targeting was achieved by engineering the dendritic cells to express Lamp2b, an exosomal membrane protein, fused to the neuron‐specific RVG peptide. But RVG peptide specifically targets neurons, microglia, and oligodendrocytes in the brain, not glioblastoma cells, it is not appropriate for the treatment of GBM. Subsequently, researchers have developed different engineered exosomes based on this model by replacing the RVG peptide, such as the Her2 peptide, and E7 peptide for different diseases.^[^ ^23^ ^]^ Inspired by these articles, we chose the angiopep‐2 peptide to replace RVG‐peptide for the first time. Angiopep‐2 (TFFYGGSRGKRNNFKTEEY, ANG) is a peptide derived from the Kunitz domains of aprotinin that specifically binds LRP‐1.^[^ ^19^ ^]^ Low‐density lipoprotein receptor protein 1 (LRP‐1) is highly expressed on brain capillary endothelial and glioma cells, and these characteristics make angiopep‐2 a promising candidate for LRP1‐mediated targeted drug delivery to glioblastoma. However, all these articles used exogenous ANG peptide, such as DSPE‐PEG‐ANG, and therefore have the possibility of degradation by various enzymes in the blood or cells. Fortunately, endogenous ANG peptide was acquired by forming a fusion gene with Lamp2b protein. Previous studies have shown that EVs can breach the BBB via transcytosis,^[^ ^16^, ^24^ ^]^ our platform demonstrated that exosomes modified with the ANG peptide have enhanced BBB penetration and tumor targeting ability. ANG‐Lamp2b plasmids were constructed according to previous research,^[^ ^18^, ^25^ ^]^ and the ANG peptide (TFFYGGSRGKRNNFKTEEY) was replaced with the RVG fragment in the pcDNA3.1 hygro vector. These plasmids included a glycosylation sequence (GNSTM), the ANG peptide at the N‐terminus of the Lamp2b protein, and hemagglutinin (HA)‐tags at the C‐terminus (**Figure** **3**a). The glycosylation sequence (GNSTM) is used to stabilize the exosome‐targeting peptides and the HA‐tag is used for western blotting to verify the identify successful fusion gene construction.^[^ ^25^ ^]^ In addition, we constructed a vector that does not contain ANG peptide for the control. MSC‐derived EVs have significant bioengineering potential because they have a strong propensity to migrate toward tumor sites,^[^ ^26^ ^]^ so we constructed a lentivirus of this plasmid to obtain more exosomes from human mesenchymal stromal cells (hMSCs). Human mesenchymal stromal cells were stably transduced with a lentivirus vector encoding this fusion protein and exosomes were purified from the culture supernatants by ultracentrifugation. We then used HA‐tagged antibodies to identify successful fusion gene construction in cells and exosomes and defined the ANG‐modified exosomes as ANG‐EXO (Figure 3b). And we identified exosomes by the recognized exosomal surface markers TSG101, Flotillin1, CD81, and CD9 and the negative marker Calnexin (Figure 3b). Similarly, we used NTA and TEM techniques to analyze the hMSC‐EXO, Lamp2b‐EXO and ANG‐EXO structures. The results showed that transfection with the ANG‐Lamp2b fusion protein did not affect the morphology (Figure 3c) or particle diameter distribution (Figure S6, Supporting Information) of the exosomes. All of these results demonstrated the successful fabrication and synthesis of ANG‐EXO.

### 2.3 Biological Functions of ANG‐EXO

ANG‐EXO have an enhanced ability to target GBM cells. Thus, we labeled exosomes with the membrane dye PKH26 (red), A172, and LN229 cells were incubated with the same concentrations of hMSC‐EXO, Lamp2b‐EXO, and ANG‐Lamp2b‐EXO at 37 °C for 6 h, and the exosome uptake was assessed by flow cytometry (Figure 3d). The quantification of mean fluorescence intensity (MFI) values in PKH26‐labeled exosomes on A172 and LN229 cells were showed in Figure S9 (Supporting Information). Compared with nontargeted peptide exosomes and normal exosomes, A172 and LN229 cells showed stronger uptake of ANG‐EXO. Additionally, we used confocal microscopy to observe the exosome uptake. The results were consistent with the flow cytometry results. There were more PKH26‐positive cells containing ANG‐EXO than the nontargeted peptide exosomes (Figure 3e,f). To evaluate the electroporation efficiency of siGPX4, we electroporated FAM‐labeled siGPX4 into PKH26‐labeled exosomes (Figure S10, Supporting Information), and the presence of the siGPX4‐exosome complex was confirmed by confocal microscopy in LN229 cells (Figure 3g). The in vitro downregulation effects of ANG‐EXO‐siGPX4 were confirmed by western blotting. These results demonstrated that the loading capacity of siRNA in 10^9^ exosomes was ≈16.6% compared with Lipofectamine 3000 (Figure S11, Supporting Information). Therefore, in subsequent electroporation experiments, we have used a concentration of 300 × 10^−9^ m for the electroporation of exosomes. In conclusion, we confirmed that ANG‐EXO had stronger tumor targeting and small interfering RNA encapsulation properties.

### 2.4 Fabrication and Biocompatibility of MNP@EXO

**Figure 4:**
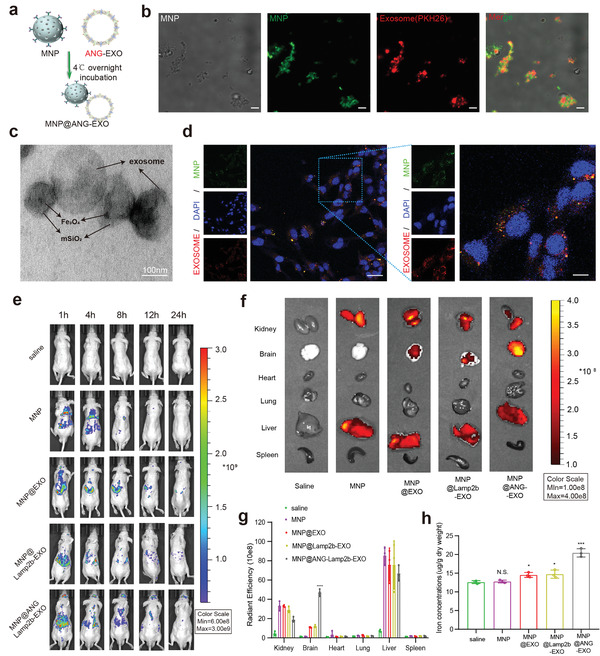
Targeting ability of MNPs@ANG‐EXO in vivo and in vitro. a) Schematic diagram of MNPs conjugated to exosomes after incubation at 4 °C overnight. b) Representative confocal microscopy images of MNPs@ANG‐EXOs, highlighting the colocalization of exosomes (red, PKH26) with the magnetic NPs (green, FITC). Brightfield and merged images are shown. Scale bar, 5 µm. c) TEM image of exosome‐conjugated magnetic nanoparticles. Scale bar, 100 nm. d) Cellular uptake of MNP@ANG‐EXO after 6 h of incubation with LN229 cells. Scale bar, 50 µm (left), 10 µm (right). e) In vivo distribution of saline, MNPs, MNP@EXO, MNP@Lamp2b‐EXO, and MNP@ANG‐EXO in orthotopic DIPG‐bearing mice at 24 h postinjection. f) Ex vivo fluorescence images of the main organs after injection under a magnetic field. g) Quantitative analysis of the fluorescence images (*n* = 3; ****p < 0.0001, compared with saline treatment group). h) ICP–MS detection of iron ions levels in the brain (*n* = 3; *p < 0.05, ***p < 0.001, compared with saline treatment group). All of the above data are shown as the mean ± SD.

After constructing engineered exosomes, we conjugated them with MNPs for subsequent drug targeting and delivery. Magnetic NPs are a widely studied biomedical nanodrug delivery system, and their nontoxicity, magnetic targeting, enhanced retention, and permeability (EPR) effect and other effects have encouraged their wide use in tumor targeting and as magnetic resonance imaging (MRI) contrast agents.^[^ ^27^ ^]^ We incubated the magnetic NP‐conjugated CD63 antibody with hMSCs exosomes in a rotational mixer at 4 °C (**Figure** **4**a), and the results showed that the magnetic NPs labeled with FITC were strongly enriched with the exosomes labeled with PKH26 in vitro (Figure 4b). The TEM image also confirmed the good compounding of exosomes with MNPs in Figure 4c. We subsequently designed an in vitro guided test of the composite material to determine the stability of the composite. The PKH26‐labeled exosomes conjugated MNPs were incubated at 4 °C overnight in phosphate‐buffered saline (PBS) buffer at either pH 7.4 or pH 5.5. After magnetic separation, the supernatant (released exosome) was added to LN229 and A172 GBM cells (Figure S12, Supporting Information). Then confocal laser scanning microscopy was used to track the intracellular localization of PKH26‐labeled exosomes (Figure S13, Supporting Information). The result showed the exosome separation of Fe3O4 only occurred at acidic buffer, the GBM cells uptake of PKH26‐labeled exosome release in acidic buffer (pH 5.5) but not in neutral buffer (pH 7.4). These results confirmed the stability of the composites, which could be separated in an acidic environment in endo/lysosome of cells. This finding also confirms that the material has had good stability under neutral pH conditions, such as in blood. In addition, we incubated exosome‐conjugated MNPs with A172 and LN229 cells and observed that GBM cells phagocytosed the MNP@EXO (Figure 4d; and Figure S14, Supporting Information). There are overlapping and nonoverlapping parts of the FITC‐labeled MNP and PKH26‐labeled exosomes, lateral evidence for the possible separation of materials under acidic intracellular conditions.

Live/dead staining of A172 and LN229 cells was used to determine cytocompatibility in DMEM and with MNPs and MNPs conjugated with exosomes (50 µg mL^−1^). Live cells were stained with calcein (green), while dead cells were stained with propidium iodide (PI) (red), and the effects of the three different culture conditions on GBM cells were analyzed by calculating the percent of red to green cells. The results showed that there was no significant difference between the three culture conditions in A172 and LN229 cells after either 24 or 72 h, and the cell survival rate in each case was greater than 97% (Figures S15 and S16, Supporting Information). Therefore, we believe that the synthesized MNPs can be sufficiently conjugated to exosomes without significant effects on cell viability and that these hMSC exosomes and MNPs can be improved and modified for subsequent drug and gene therapy delivery.

### 2.5 MNP@ANG‐EXO Tumor Targeting

To further verify the BBB penetration and tumor targeting ability of the material in vivo, we constructed a human‐derived tumor xenograft GBM model using LN229 GBM cells to validate the tumor targeting ability of these NPs. First, 5*10^5^ LN229 cells were diluted in 10 µL of PBS and injected into the right frontal lobe of each male athymic nude mouse. We verified successful tumor implantation with a small animal fluorescence imaging device on day 7. After confirming that the tumors were approximately the same size, we placed a helmet with permanent magnets (made of neodymium iron boron) on each mouse to enrich the vascular NPs into the heads of the mouse for 30 min after tail veins injection. Saline, FITC‐labeled MNPs, MNP@EXO, MNP@Lamp2b‐EXO, or MNP@ANG‐lamp2b‐EXO were injected through the mouse tail veins on days 7, 10, and 13 (Figure S17, Supporting Information). The nude mice were imaged at different time points post intravenous (i.v.) injection of various nanoagents on day 7 (Figure 4e). The results shown this inorganic/organic complex significantly prolonged the blood circulation for a long time, and the angiopep‐2 peptide‐modified exosome group was more enriched in the brain and had a long half‐life time. The MNP@ANG‐Lamp2b‐EXO‐treated mice clearly displayed stronger fluorescence in the brain after 24 h postinjection. The stronger BBB penetration and brain accumulation ability of MNP@ANG‐Lamp2b‐EXO were confirmed by ex vivo imaging of the main organs of mice sacrificed 12 h postinjection on day 13, as shown in Figure 4f,g. In addition, immunofluorescence staining of frozen mouse brain sections was performed at 13 days to observe the exosome distribution. As shown in Figure S18 (Supporting Information), MNP@ANG‐Lamp2b‐EXO had the highest enrichment in the brain tumor region. In addition, ICP–MS was used to detect iron ions levels in the brain, as shown in Figure 4h. The results confirmed that the MNP@ANG‐Lamp2b‐EXO group had the highest amount of iron ions in the brain. Therefore, we believe that our constructed NPs have a strong ability to cross the BBB and reach the tumor site.

### 2.6 Enhanced Ferroptosis Caused by MNP@BQR@ANG‐EXO‐siGPX4 In Vitro

**Figure 5:**
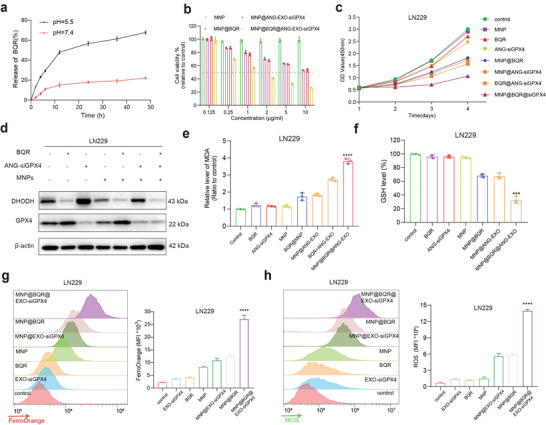
In vitro enhancement of ferroptosis by MNPs@BQR@ANG‐EXO‐siGPX4. a) Percent of BQR released from the MNPs in solutions with different pH values. b) Viability of LN229 cells after coculture with different concentrations of MNPs, MNP@BQR, MNP@ANG‐EXO‐siGPX4, or MNP@ANG‐EXO‐siGPX4@BQR for 48 h. c) Growth curve based on the OD450 using a CCK‐8 assay in cells after coculture with different NPs in LN229 cells. d) Western blot showing the protein expression of DHODH and GPX4 in LN229 cells after the addition of different NPs. e) MDA levels detected in LN229 cells after coculture with different NPs (*n* = 3 and were normalized to the level in the control group; ****p < 0.0001, compared with control group). f) GSH levels in LN229 cells treated with different NPs for 48 h (*n* = 3 and were normalized to the level in the control group; ***p <0.001, compared with control group). g) Flow cytometry analysis of Fe^2+^ (FerroOrange staining) in LN229 cells incubated with different NPs. And the quantification of mean fluorescence intensity (MFI) values in FerroOrange on LN229 cells (*n* = 3 and were normalized to the level in the control group; ****p < 0.0001, compared with control group). h) Flow cytometry analysis of ROS (DCFH‐DA staining) in LN229 cells incubated with different NPs. And the quantification of mean fluorescence intensity (MFI) values in ROS on LN229 cells (*n* = 3 and were normalized to the level in the control group; ****p < 0.0001, compared with control group). All of the above data are shown as the mean ± SD.

We introduced the latest concepts of ferroptosis into our platform. Ferroptosis is a new form of cell death that was discovered in 2012.^[^ ^6d^ ^]^ As research continues in this area, specific mechanisms and key proteins continue to be discovered by researchers. Recent studies have determined the latest defense mechanism of the ferroptosis‐DHODH system, which is a novel defense system located in mitochondria that can regulate ferroptosis independent of the GSH pathway. DHODH is a ferroptosis defense axis independent of the classical GPX4 signaling pathway in mitochondria (Figure S19, Supporting Information). When GPX4 is inhibited, DHODH compensates by elevating its expression to counteract cell ferroptosis (Figures S3 and S20, Supporting Information).^[^ ^9^ ^]^ Therefore, we added siGPX4 to the exosomes and an inhibitor of DHODH (BQR) to the MNPs. In addition, Fe_3_O_4_‐based core NPs have been shown to have the ability to induce ferroptosis themselves.^[^ ^28^ ^]^ Thus, by promoting these three effects, this system has a significant ability to promote ferroptosis in GBM cells. We identified normal human astrocytes (NHAs), U87MG, U251MG, U118MG, A172, and LN229 cells by western blotting and found that all GBM cells had high expression levels of DHODH, GPX4, and LRP‐1 compared with NHAs, and we chose A172 and LN229 for our experiments (Figure S2, Supporting Information). In addition, tumor cells have an acidic intracellular microenvironment in endo/lysosomes; therefore, we simulated the ability of the drug to be released from mesoporous silica at different pH values in vitro. The results showed that BQR reached 68% release in acidic medium at pH 5.5 after 48 h (**Figure** **5**a).

To evaluate the antitumor capacity of the NPs in vitro, we evaluated the IC50 values of different composite NPs. Compared with free MNPs, MNP@BQR, or MNP@ANG‐EXO‐siGPX4, the viability of LN229 cells was dramatically reduced after treatment with lower concentrations of MNP@BQR@ANG‐EXO‐siGPX4, giving an IC50 of ≈2 µg mL^−1^ (Figure 5b). In addition, we examined the IC50 values of the DHODH inhibitor BQR. The IC50 values of BQR in A172 and LN229 cells were 44.25 and 24.24 × 10^−9^ m, respectively (Figure S21, Supporting Information). From these data, we determined the concentration of each NPs required for cellular experiments (the concentrations of MNPs, MNP@BQR, MNP@ANG‐EXO‐siGPX4, and MNP@BQR@ANG‐EXO‐siGPX4 were each 5 µg mL^−1^; and that of ANG‐EXO‐siGPX4 was 300 × 10^−9^ m). The growth curve based on the results of the CCK‐8 assay were used to analyze the effects of different combinations of particles or drugs on cell growth. No significant effect on cell growth was observed in the groups treated with BQR, ANG‐EXO‐siGPX4, and NPs (5 µg mL^−1^) alone, but the combining two of BQR, siGPX4, or MNPs inhibited GBM cell growth to some extent. Combining all three led to the strongest tumor growth inhibition effects (Figure 5c). Similarly, the western blot results suggested that the combination of MNPs, BQR, and siGPX4 reduced both intracellular DHODH and GPX4 levels (Figure 5d). A lipid peroxidation MDA assay kit was used to observe lipid oxidation levels, as MDA indirectly reflects the extent of ferroptosis. Lipid oxidation occurs when cells undergo oxidative stress, and MDA is a natural product of lipid oxidation in living organisms. When A172 and LN229 cells were stimulated with the composite NPs for 48 h, the overall cellular MDA levels increased by ≈3–4 fold compared with normal cells and those treated with drug or siGPX4 alone (Figure 5e; and Figure S22, Supporting Information). Interestingly, the intracellular GSH levels showed the opposite trend (Figure 5f). Because BQR and siGPX4 increased intracellular GSH depletion, thus promoting cellular ferroptosis. FerroOrange (Dojindo, Japan) is a novel fluorescent probe that allows fluorescent imaging of Fe^2+^ in living cells. We detected intracellular Fe^2+^ by flow cytometry, and the results showed that the intracellular Fe^2+^ concentration gradually increased with the addition of MNPs to A172 and LN229 cells, and the highest level of Fe^2+^ was reached after treatment with MNP@BQR@ANG‐EXO‐siGPX4 (Figure 5g; and Figure S23, Supporting Information). This experiment also demonstrated that MNPs alone can increase the intracellular Fe^2+^ content to a certain extent. Moreover, a DCFH‐DA probe was used to detect intracellular ROS levels. As shown in Figure 5h, treatment with MNPs, BQR, or siGPX4 alone increased intracellular ROS levels to some extent, but the ROS level reached its maximum in MNP@BQR@ANG‐EXO‐siGPX4‐treated cells (Figure 5h; and Figure S24; Supporting Information). In conclusion, we believe that the application of MNPs, BQR, or siGPX4 alone does not lead to ferroptosis because of intracellular compensatory mechanisms. Interestingly, using these strategies in combination can disable the intracellular compensatory mechanism and promote ferroptosis of GBM cells.

### 2.7 Antitumor Effects In Vivo

**Figure 6:**
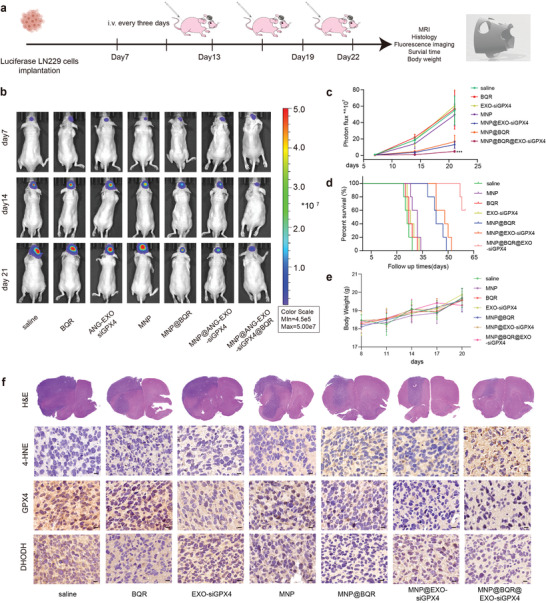
Antitumor efficiency of MNP@BQR@ANG‐EXO‐siGPX4. a) Timeline and 3D printed mouse helmet model schematic of the animal experiment. b) Luminescence images of orthotopic LN229‐Luc^+^ human GBM tumor‐bearing nude mice following different treatments monitored on days 7, 14, and 21. c) Quantitative analysis of the luminescence images (*n* = 5; ***p < 0.001, compared with saline treatment group). d) Survival curves of the mice in the different groups, *n* = 5. e). Mouse body weight changes during different treatments, *n* = 5. f) Representative H&E images and 4‐HNE, GPX4 and DHODH immunochemistry images of the xenograft GBM tumors after the indicated treatment. Scale bar, 10 µm. All of the above data are shown as the mean ± SD.

We constructed a human‐derived tumor xenograft GBM model using Luci^+^ LN229 cells to evaluate the antitumor effects of the NPs (**Figure** **6**a). Nude mice were randomly grouped at day 7 after tumor implantation and subsequently injected with different concentrations of NPs via the tail vein every 3 days in addition to magnetic targeting with the 3D printed mouse helmet model. As shown from the bioluminescence assay, MNP@BQR@ANG‐EXO‐siGPX4 treatment exerted the strongest inhibitory effect on tumor regrowth (Figure 6b,c). Similarly, the Kaplan–Meier survival curve of the nude mice showed that survival was longest in the group injected with MNP@BQR@ANG‐EXO‐siGPX4, where three mice were still alive on day 60 (Figure 6d). There was no significant difference in mouse body weights after tail vein injection of different NPs on day 20, suggesting that the NPs were well tolerated throughout the experiment (Figure 6e). Hematoxylin and eosin (H&E) staining revealed the location and size of GBM in mice brains. We used 4‐hydroxynonenal (4‐HNE; a lipid peroxidation marker), DHODH and GPX4 immunohistochemistry to verify the extent of ferroptosis, and MNP@BQR@ANG‐EXO‐siGPX4 markedly increased 4‐HNE staining and decreased DHODH and GPX4 staining (Figure 6f). In addition, LRP‐1 was highly expressed in tumor tissues compared with normal tissues as stated in the literature review (Figure S25, Supporting Information).^[^ ^19^ ^]^ Numerous articles have reported that MNPs can increase the T2 images of MRI.^[^ ^19^, ^29^ ^]^ Exosome‐conjugated magnetic NPs can be guided by a magnetic field to become enriched in the brain, and the particles can be detected by mouse cranial MRI. Through in vivo MRI of intracranial GBM, the GBM location was determined and NP signals were observed in T2‐weighted signal (Figure S26, Supporting Information). We used H&E staining of the tissue of the mice to verify the biocompatibility of the particles, and the results showed no significant organ damage, demonstrating that this therapy is safe and biocompatible (Figure S27, Supporting Information). In addition, we used blood routine and blood biochemical to confirm that nanoparticle injection did not cause a significant inflammatory response and that there were no significant abnormalities in the liver or kidney function of the mice (Figure S28, Supporting Information). Thus, our animal studies showed that this nanoplatform has the potential to penetrate the BBB, inhibit glioma growth and facilitate MRI to some extent.

## 3 Conclusions

In summary, we designed a therapeutic platform based on engineered exosome‐conjugated magnetic nanoparticles that can enhance ferroptosis in GBM. The results demonstrated that our platform possesses the following advantages. 1) We achieved the first synergistic treatment of GBM through the triple action of disrupting DHODH, the GPX4 ferroptosis defense mechanism and MNP‐mediated Fe^2+^ release. Combined with the Fe_3_O_4_ particles themselves, after loading the DHODH inhibitor and siGPX4, this platform showed powerful ferroptosis promotion in GBM. 2) Angiopep‐2 peptide‐modified exosomes not only possess the ability to penetrate the BBB but also target GBM cells by recognizing LRP‐1 receptors, and endogenous angiopep‐2 peptide was acquired by forming a fusion gene with Lamp2b protein. 3) This conjugated system can achieve local targeting under the action of an external magnetic field. 4) The engineered exosome‐conjugated MNPs have good biocompatibility and safety. 5) We designed a corresponding wearable magnetic helmet for mice through 3D printing technology in this study. Naturally, this NP platform can be modified accordingly for other malignant solid tumors. The permanent magnet can even be improved to produce an alternating magnetic field and achieve location‐specific targeting for precision treatment. Moreover, we can improve the platform‐loaded drugs for other diseases. We hope that our work will provide a new strategy for the treatment of GBM and broaden the prospects for precise diagnosis and treatment of GBM in the future.

## 4 Experimental Section

### NP Synthesis

In a typical synthesis protocol, iron oxide NPs were synthesized according to a previous method. First, an iron‐oleate complex was synthesized with iron chloride (FeCl_3_·6H_2_O) and sodium oleate. Next, the synthesized iron‐oleate complex and oleic acid were dissolved in 1‐octadecene. Then, the reaction temperature was increased to 320 °C and maintained for 30 min. After the reaction, the products were rapidly cooled to room temperature, and ethanol was added. Finally, the magnetic NPs were precipitated by centrifugation.

### Synthesis of Fe_3_O_4_@mSiO_2_ and FITC Labeling of MNPs

Fe_3_O_4_@mSiO_2_ NPs were prepared according to a previous study. A chloroform solution containing Fe_3_O_4_ NPs was mixed with water and cetyltrimethylammonium bromide (CTAB). Then, the mixture was vigorously stirred at 60 °C to evaporate the chloroform. Next, 20 mL of an aqueous Fe_3_O_4_ solution (1 mg mL^−1^) was added to 2 m sodium hydroxide and distilled water and heated to 70 °C. Then, silicon tetraacetate (TEOS) was added to the mixture. After 15 min (3‐aminopropyl) trimethoxysilane (APTMS) was placed into the reaction system, which was stirred for 1.5 h. Finally, the amino‐functionalized Fe_3_O_4_ @mSiO_2_ NPs were centrifuged, washed, and redispersed in water. To generate FITC‐labeled Fe_3_O_4_ @mSiO_2_ NPs, FITC‐conjugated APTMS (FITC‐APTMS) was first synthesized by reaction of the FITC with APTMS in ethanol. Then, the FITC‐APTMS solution was added before TEOS according to the same procedure described above for Fe_3_O_4_ @mSiO_2_.

### Anti‐CD63 Conjugated Fe_3_O_4_@mSiO_2_ NPs

To obtain anti‐CD63 antibody‐conjugated Fe_3_O_4_ @mSiO_2_ NPs, NHS/EDC crosslinking chemistry was employed. The anti‐CD63 antibody was activated by adding NHS and EDC, and then amino‐functionalized Fe_3_O_4_ @mSiO_2_ NPs were added to the mixture for incubation overnight at 4 °C. Subsequently, the generated anti‐CD63 antibody (Abcam, ab231975; Santa Cruz Biotechnology, sc‐5275) conjugated to the Fe_3_O_4_ @mSiO_2_ NPs was centrifuged and washed with PBS several times. For characterization, the morphology of the MNPs was observed by SEM (S‐4800, Hitachi, Japan) and TEM (JEM‐2100, Japan). The morphology of the Fe_3_O_4_ NPs was observed by (Talos F200X, Thermo Fisher). XRD patterns were obtained on a Bruker D8 Advance Powder Diffractometer equipped with a Cu K*α* sealed tube. FTIR spectra were recorded with a Fourier transform infrared spectrometer (Tensor II, Bruker, USA). Magnetization curves were recorded using a MicroMag model 2900 alternating gradient magnetometer (Princeton Measurements Cooperation).

### Drug Loading

The DHODH inhibitor Brequinar (BQR) was purchased from MCE (HY‐108325) and Selleck (S6626) and used according to the instructions. Fe_3_O_4_@SiO_2_‐NH2 NPs were mixed with BQR in PBS (pH  =  7.4). After stirring overnight, the BQR‐loaded sample was collected by centrifugation at 13 000 rpm for 15 min. The residual BQR in the supernatant was measured by UV–vis spectrophotometry. The BQR loading efficiency (LE%) was calculated as follows:$\mathrm{LE}\%=\frac{\mathrm{original}\mathrm{BQR}-\mathrm{BQR}\mathrm{in}\mathrm{supernatant}}{\mathrm{original}\mathrm{BQR}}*100\%$.

### Cell Lines and Culture

The human GBM cell lines U87, U251, and A172 were purchased from the Chinese Academy of Sciences Cell Bank (Shanghai, China). The human GBM cell lines LN229 and U118 were purchased from the American Type Culture Collection. These cells cultured in Dulbecco's modified Eagle's medium (DMEM; Thermo Fisher Scientific). Normal human astrocytes (NHAs) were obtained from Lonza Group, Ltd. (USA) and cultured in the provided astrocyte growth media supplemented with insulin, ascorbic acid, GA‐1000, L‐glutamine, and 5% fetal bovine serum (FBS). hMSCs were obtained from the Lonza Group, Ltd. (USA) and cultured in human MesenCult medium (STEMCELL Technologies, Vancouver). The STR profiles of LN229 and A172 cell lines were shown in Figures S29 and S30 (Supporting Information).

### Western Blotting

Protein was extracted from cells and exosomes using RIPA lysis buffer containing protease inhibitor cocktail (Sigma‐Aldrich). The following primary antibodies were used: *β*‐actin (Proteintech, 66009‐1), GPX4 (Abcam, ab125066), DHODH (Proteintech, 14877‐1), HA (Proteintech, 51064‐2), and LRP‐1 (Abcam, ab92544).

### Exosome Purification, Characterization, and Analysis

Cell culture medium was centrifuged at 500 g for 10 min, then the supernatant was centrifuged at 20 000 g for 20 min. The supernatant was transferred to a fresh tube, filtered through a 0.22 µm filter and pelleted by ultracentrifugation (Beckman Optima L‐100 XP, Beckman Coulter) at 100 000 g for 70 min. Exosome pellets were washed in a large volume of PBS and recovered by centrifugation at 100 000 g for 70 min. The particle concentration and size distribution of the purified exosomes were determined using NTA in ZetaView (Particle Metrix). The morphology and size of the exosomes were determined using SEM. Purified exosomes were characterized by western blotting using antibodies against TSG101 (Abcam, ab125011), CD9 (System Biosciences, ExoAB‐CD9A‐1), Calnexin (Cell Signaling Technology, 2679), Flotillin1 (Proteintech, 15571‐1), and CD81 (Proteintech, 66866‐1).

### Vector Construction and Lentivirus Preparation

pcDNA GNSTM‐3‐RVG‐10‐Lamp2B‐HA was constructed with reference to Addgene's (Watertown, MA) plasmid (Plasmid #71294; http://www.addgene.org/71294/). The angiopep‐2 peptide (TFFYGGSRGKRNNFKTEEY) was replaced with the RVG fragment in the pcDNA3.1 hygro vector. The recombinant plasmids were transformed into hMSCs using Lipofectamine 3000 transfection reagent (Invitrogen, USA) according to the manufacturer's instructions. Lentivirus construction was carried out by GeneChem Biologicals Inc. (Shanghai, China).

### Exosome Loading

A total of 10^9^ exosomes (measured by NTA) were mixed with 1.5 µg siGPX4(siGPX4#1: 5’‐GTGGATGAAGATCCAACCCAA‐3’; siGPX4#2: 5’‐GCACATGGTTAACCTGGACAA‐3’) in electroporation buffer (PBS pH 7.4). Exosomes were electroporated using a single 4 mm cuvette and a Lonza Nucleofector 2B system. After electroporation, the exosomes were treated with RNase to remove any siRNAs that might be bound to the exosome membrane. Then, the exosomes were diluted with cold PBS and centrifuged at 100 000 g for 70 min to remove unbound siGPX4.

### In Vitro Exosome Binding and Targeting Studies

Exosomes were labeled with PKH26 (red) membrane dye (Sigma‐Aldrich) according to the manufacturer's instructions. For the composite NPs, the MNPs mentioned above and extracted exosomes were incubated overnight at 4 °C at a 1:1 ratio. The obtained compound was separated and washed with PBS under a magnetic stand. Conjugation was verified by fluorescently labeling the MNPs with FITC and the exosomes with PKH26.

### In Vitro Cellular Uptake

A172 and LN229 cells were seeded in a 6‐well plate (Corning). When cell confluence reached 70–90%, PKH26‐labeled hMSC exosomes, Lamp2b‐EXO, or ANG‐EXO in DMEM medium were added for incubation at 37 °C for 6 h. Then, the uptake efficiency of the exosomes was detected by confocal laser scanning microscopy (Leica SP8) and flow cytometry (BD Accuri C6 Flow Cytometer).

### Live/Dead Staining

To visually monitor the number of live and dead GBM cells, live/dead staining (Beyotime Biotechnology, China) was performed according to the manufacturer's instructions. Briefly, A172 and LN229 cells were cultured in 48‐well plates with DMEM medium, MNPs, or MNP@exosomes for 24 or 72 h, and the dyes calcein AM and PI were cultured with cells for an additional 30 min at 37 °C. Images of live and dead cells were captured by confocal microscopy (Leica SP8).

### Animal Studies

Four‐week‐old male athymic nude mice (SLAC Laboratory Animal Center, Shanghai, China) were bred under specific pathogen‐free conditions at 24 °C on a 12 h day‐night cycle. All experimental procedures were approved by the Research Ethics Committee of Shandong University and the Ethics Committee of Qilu Hospital (Shandong, China). For intracranial GBM xenografts, 5 × 10^5^ LN229 cells were diluted in 10 µL of PBS and injected into the right frontal lobe of each mouse. The permission number of animal experiment ethical approval is DWLL‐2021‐40.

### Magnetic Targeting

3D printing technology was used to construct a mouse helmet based on photosensitive resin material, and a neodymium iron boron magnet with a diameter of ≈8 × 2 mm^2^ (2000 gs) was placed into the top of the helmet (Video S1, Supporting Information). The size of the helmet refers to the head setting of the mouse. The mouse wore helmet for 30 min after each injection.

### In Vivo Binding and Release Studies

FITC‐labeled MNPs and PKH26‐labeled exosomes were used for in vivo experiments. hMSC exosomes, MNP@exo, MNP@Lamp2b, and MNP@ANG‐EXO (10 mg kg^−1^ body weight in 200 µL of PBS) were intravenously injected into nude mice via the tail vein with magnetic targeting. After 3 consecutive days of injection, the brains were harvested and sectioned into 10 µm frozen sections. The percent of PKH26‐labeled exosomes bound to the total number of cells was calculated.

### Ex Vivo Binding and Release Studies

FITC‐labeled MNPs and different exosomes were used for ex vivo experiments. Normal saline, MNPs, MNP@EXO, MNP@Lamp2b‐EXO, and MNP@ANG‐EXO (10 mg kg^−1^ body weight in 200 µL of PBS) were intravenously injected into the nude mice via the tail vein with magnetic targeting for 30 min. The organs were harvested and examined via bioluminescence imaging using In Vivo Imaging System (IVIS) (Spectrum, Perkin‐Elmer; Waltham, MA).

### Lipid Peroxidation Assessment

Cell lysates were centrifuged at 12 000 g for 10 min, and the supernatant was collected. Total protein content was measured using a BCA protein detection kit. Lipid peroxidation levels were detected using an MDA assay kit (Beyotime) according to the manufacturer's instructions.

### Iron Staining

A172 and LN229 cells (1 × 10^5^) were seeded in 6‐well plates with medium for 48 h of preculture with different NPs. After cleaning each well 3 times with HBSS, 1 × 10^−6^ m FerroOrange (Dojindo, Japan) in HBSS was added for 30 min of incubation at 37 °C. The content of Fe^2+^ was detected by flow cytometry (BD Accuri C6 Flow Cytometer).

### Measurement of Intracellular ROS

ROS levels were analyzed with a ROS Assay Kit‐Highly Sensitive DCFH‐DA (Dojindo, Japan). Briefly, induced cells were washed twice with HBSS, and the highly sensitive DCFH‐DA working solution was added for 30 min of incubation at 37 °C. Flow cytometry data were collected with a BD Accuri C6 flow cytometer.

### In Vivo Antitumor Effects

When the mice tumors reached a volume of 5 × 10^6^ radiance according to a fluorescence imaging device, seven groups with five mice in each group were randomly created: saline (200 µL); BQR (10 mg kg^−1^ body weight); ANG‐EXO‐siGPX4 (1.0 × 10^11^ particles in 200 µL); MNP, MNP@BQR, MNP@ANG‐EXO‐siGPX4, and MNP@BQR@ANG‐EXO‐siGPX4 (10 mg kg^−1^ body weight). The appropriate substance was injected into the mouse tail veins from day 7 with magnetic targeting followed by subsequent injections every 3 days until day 22. The size of each tumor was imaged with a fluorescence imaging device (Perkin‐Elmer; Waltham, MA). The body weights of the mice were measured 1 day after tail vein injection of the drug. Animals that displayed symptoms of complications (such as a severe hunchback posture, apathy, decreased motion, or activity, leg dragging, unkempt fur, or a drastic loss in body weight) were sacrificed by cervical dislocation. Subsequently, the mice were perfused, and the brain and tissues were subjected to follow‐up H&E and immunohistochemical (IHC) staining.

### Statistical Analysis

Each experiment was repeated independently at least three times. All data were presented as the mean ± standard deviation (*n* = 3 or *n* = 5) and were normalized to the respective control group. Student's *t*‐test was used for statistical analysis of two groups, whereas differences among multiple groups were evaluated using one‐way analysis of variance. Survival data were determined for every group by the Kaplan–Meier method and compared by the log‐rank (Mantel–Cox) test. The *P* value was calculated with the software GraphPad Prism 7. Significant differences are indicated as *P < 0.05, ** *P* < 0.01, *** *P* < 0.001, and **** *P* < 0.0001.

## Conflict of Interest

The authors declare no conflict of interest.

## Author Contributions

B.L. and X.C. contributed equally to this work. B.L. conceived and conducted the experiments, performed the analyses, and wrote the manuscript. X.C. involved in the design of materials and wrote the manuscript. J.D. gave technical guidance on material synthesis. S.Z. was involved in the painting of the diagram. W.Q., R.Z., Z.P., S.Z., Q.G., Y.Q., and W.W. contributed to the animal experiments. L.D. and S.N. contributed to the concept promotion and revised the manuscript. Y.S., H.X., H.L., and G.L. contributed to the conceptualization and resources, supervised the research, and revised the manuscript.

## Supporting information

Supporting Information

Supplemental Video 1

## Data Availability

The data that support the findings of this study are available from the corresponding author upon reasonable request.
