# Supplementary material for: Synchronous Disintegration of Ferroptosis Defense Axis via Engineered Exosome‐Conjugated Magnetic Nanoparticles for Glioblastoma Therapy
Source: Adv Sci (Weinh). 2022 May 4;9(17):2105451. doi: 10.1002/advs.202105451 (PMC9189685; doi:10.1002/advs.202105451)
Supplement: Supplementary file 1 — Supporting Information [file ADVS-9-2105451-s002.pdf]

ADVANCED SCIENCE
Open Access

Supporting Information

for *Adv. Sci.*, DOI 10.1002/advs.202105451

Synchronous Disintegration of Ferroptosis Defense Axis via Engineered Exosome-Conjugated Magnetic Nanoparticles for Glioblastoma Therapy

Boyan Li, Xin Chen, Wei Qiu, Rongrong Zhao, Jiazhi Duan, Shouji Zhang, Ziwen Pan, Shulin Zhao, Qindong Guo, Yanhua Qi, Wenhan Wang, Lin Deng, Shilei Ni, Yuanhua Sang, Hao Xue*, Hong Liu* and Gang Li**

Supporting Information

Synchronous Disintegration of Ferroptosis Defense Axis via Engineered Exosome-conjugated Magnetic Nanoparticles for Glioblastoma Therapy

Boyan Li, Xin Chen, Wei Qiu, Rongrong Zhao, Jiazhi Duan, Shouji Zhang, Ziwen Pan, Shulin Zhao, Qindong Guo, Yanhua Qi, Wenhan Wang, Lin Deng, Shilei Ni, Yuanhua Sang, Hao Xue*, Hong Liu*, Gang Li**

Affiliations

B. Li, W. Qiu, R. Zhao, S. Zhang, Z. Pan, S. Zhao, Q. Guo, Y. Qi, L. Deng, S. Ni, Prof. H. Xue, Prof. G. Li

Department of Neurosurgery

Qilu Hospital, Cheeloo College of Medicine and Institute of Brain and Brain-Inspired Science, Shandong University

Jinan 250012, P.R. China

Email: xuehao@sdu.edu.cn(H. Xue); dr.ligang@sdu.edu.cn (G. Li)

X. Chen, J. Duan, W. Wang, Prof. Y. Sang, Prof. H. Liu

State Key Laboratory of Crystal Materials

Shandong University

Jinan 250100, P.R. China

E-mail: sangyh@sdu.edu.cn (Y. Sang); hongliu@sdu.edu.cn (H. Liu)

Prof. H. Liu

Institute for Advanced Interdisciplinary Research (IAIR)

University of Jinan

Jinan 250022, P. R. China

E-mail: hongliu@sdu.edu.cn (H. Liu)

Keywords: exosomes, ferroptosis, glioblastoma, magnetic nanoparticles, blood–brain barrier

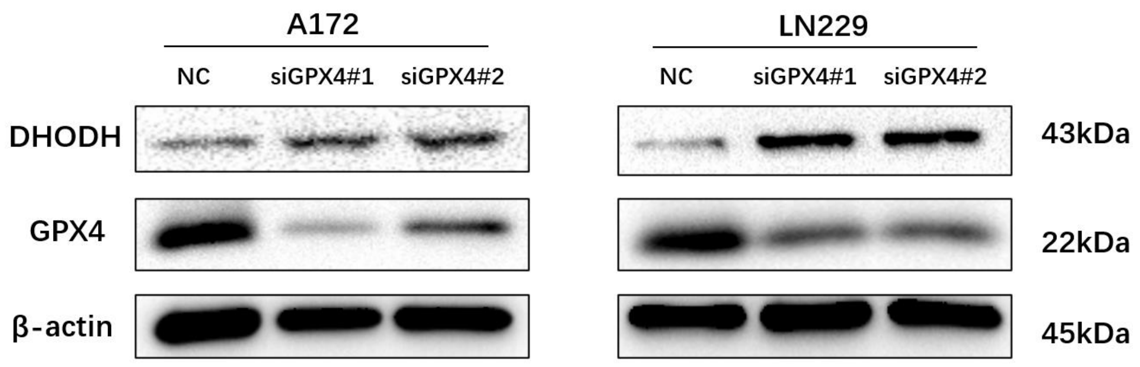

Figure S1. Western blot shows the siGPX4 transfection efficiency of A172 and LN229 cells the consequent expression of DHODH protein. And we use the siGPX4#1 for subsequent experiments

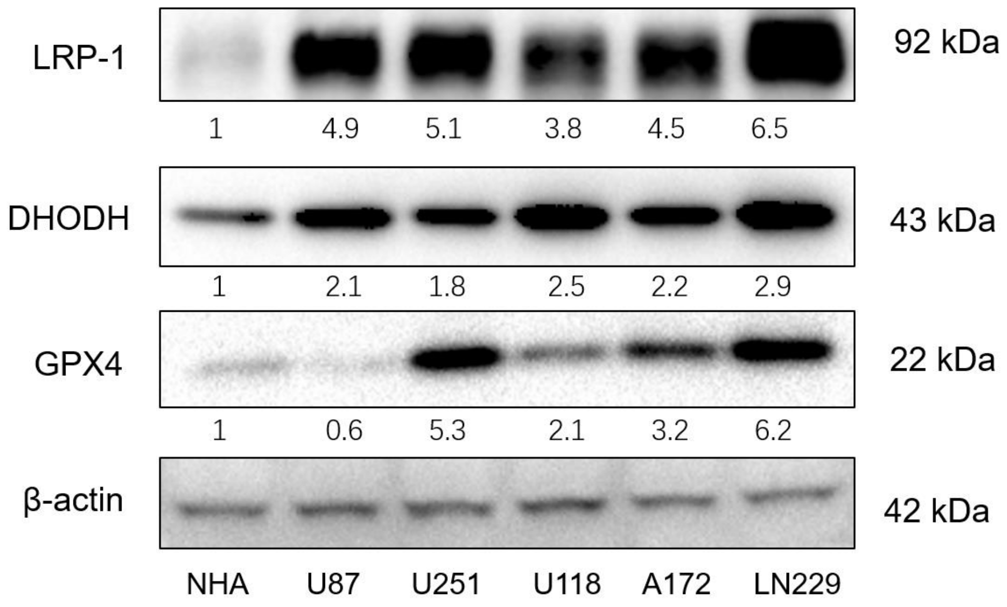

Figure S2. Western blot analysis of levels of the LRP-1, DHODH and GPX4 protein from NHA, U87MG cells, U251MG, U118MG, A172 and LN229 cells.

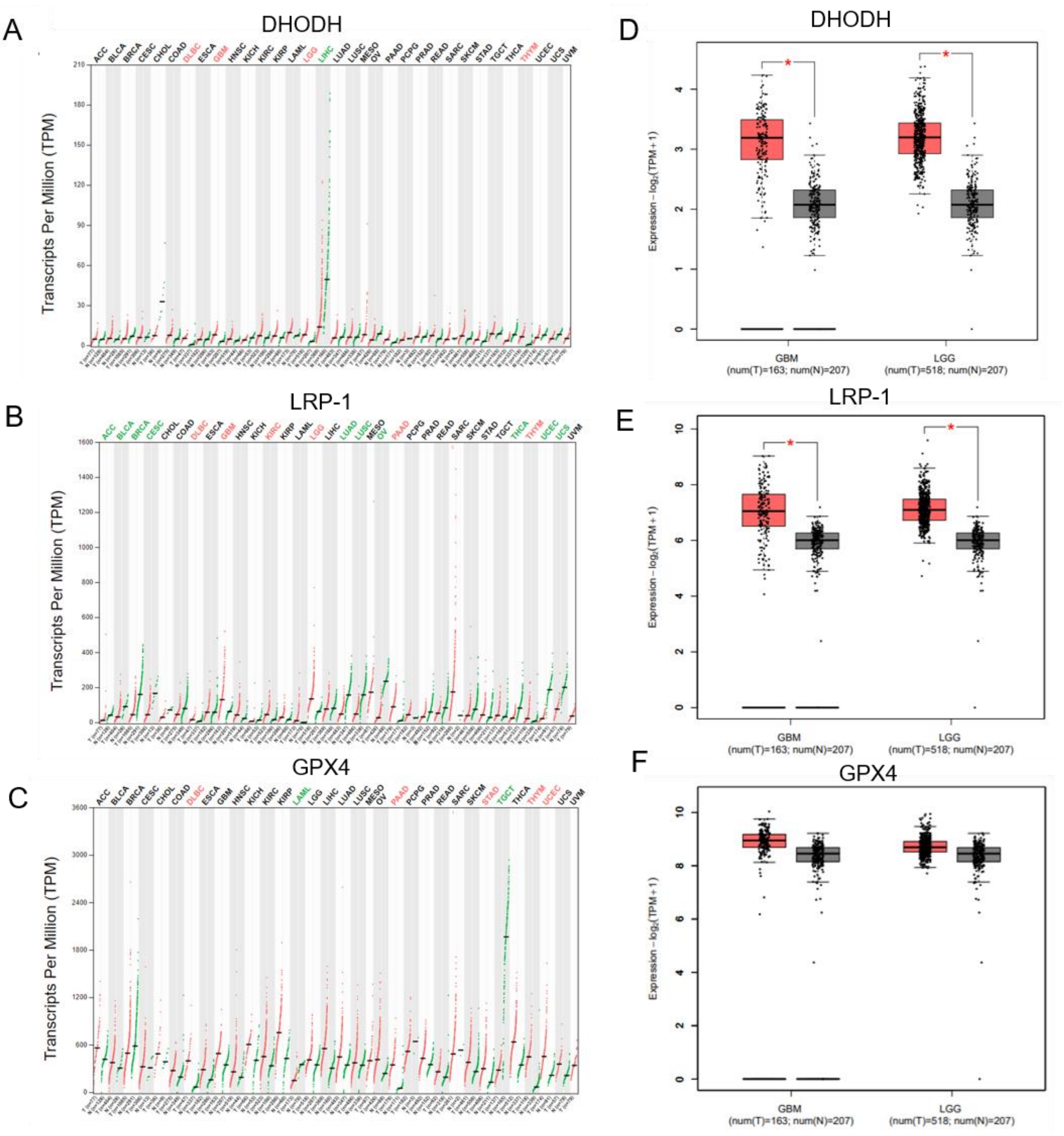

Figure S3. A-C) DHODH, LRP-1 and GPX4 RNA expression (log_2 TPM) in all tumor samples and paired normal tissues as determined from the GEPIA database; D-F) DHODH, LRP-1 and GPX4 RNA expression (log_2 TPM+1) in GBM and low-grade glioma (LGG) as determined from the GEPIA database. Normal brain tissues (n = 207) and glioma (GBM, n = 163; LGG, n =518).

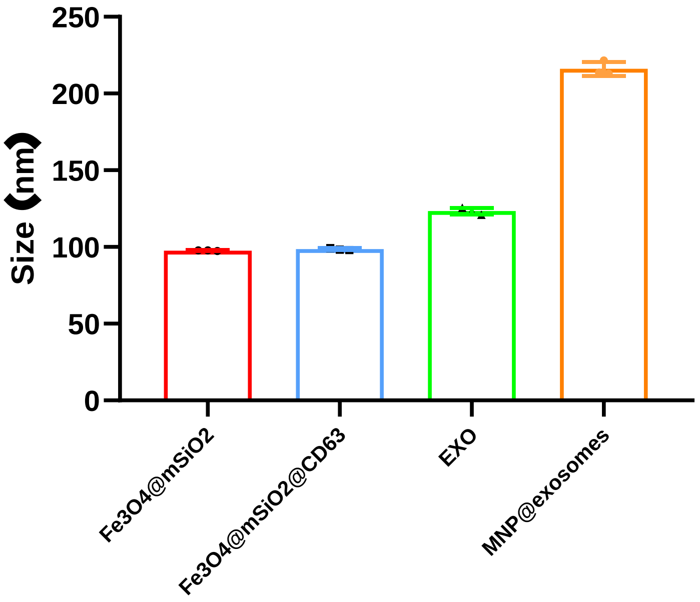

Figure S4. Size distribution of Fe_3O_4@mSiO_2, Fe_3O_4@mSiO_2@CD63, exosomes and MNP@exosomes determined by NTA (n=3 independent samples). Data are presented as mean ± s.d.

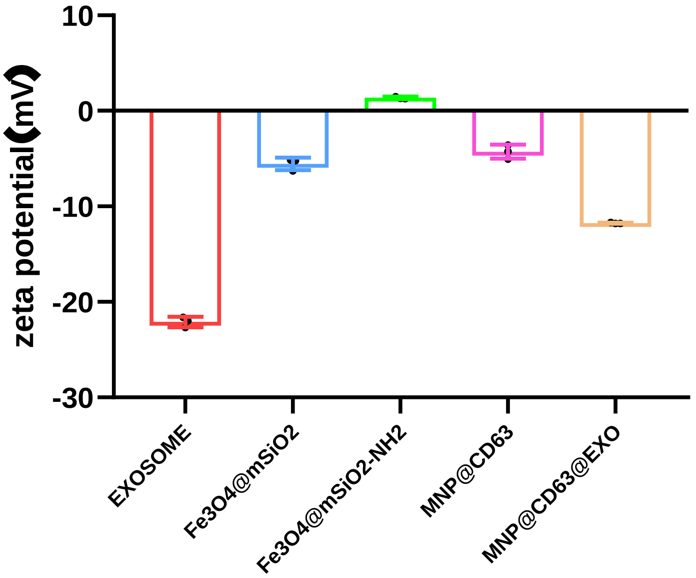

Figure S5. Zeta potential of the nanoparticles before and after the stepwise modification (n=3 independent samples). Data are presented as mean ± s.d.

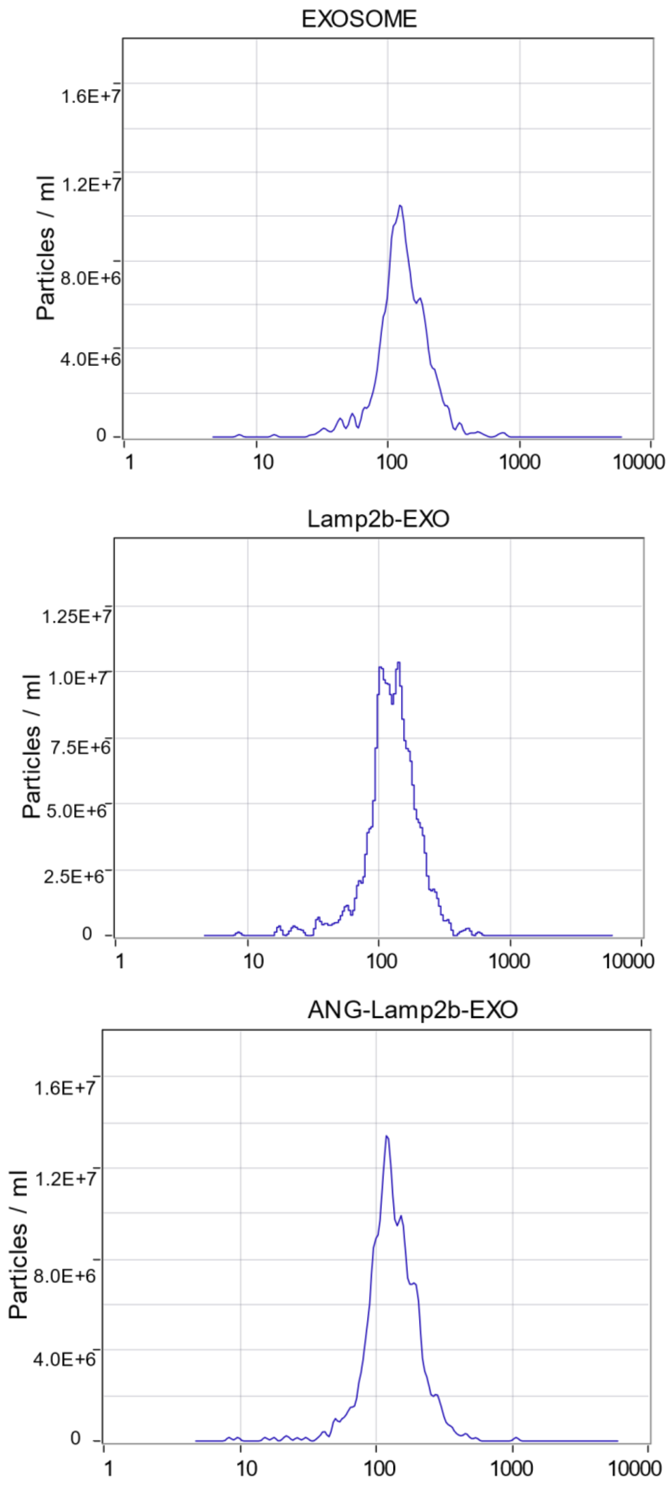

Figure S6. The nanoparticle tracking analysis (NTA) of exosome, Lamp2b-EXO and ANG-Lamp2b-EXO.

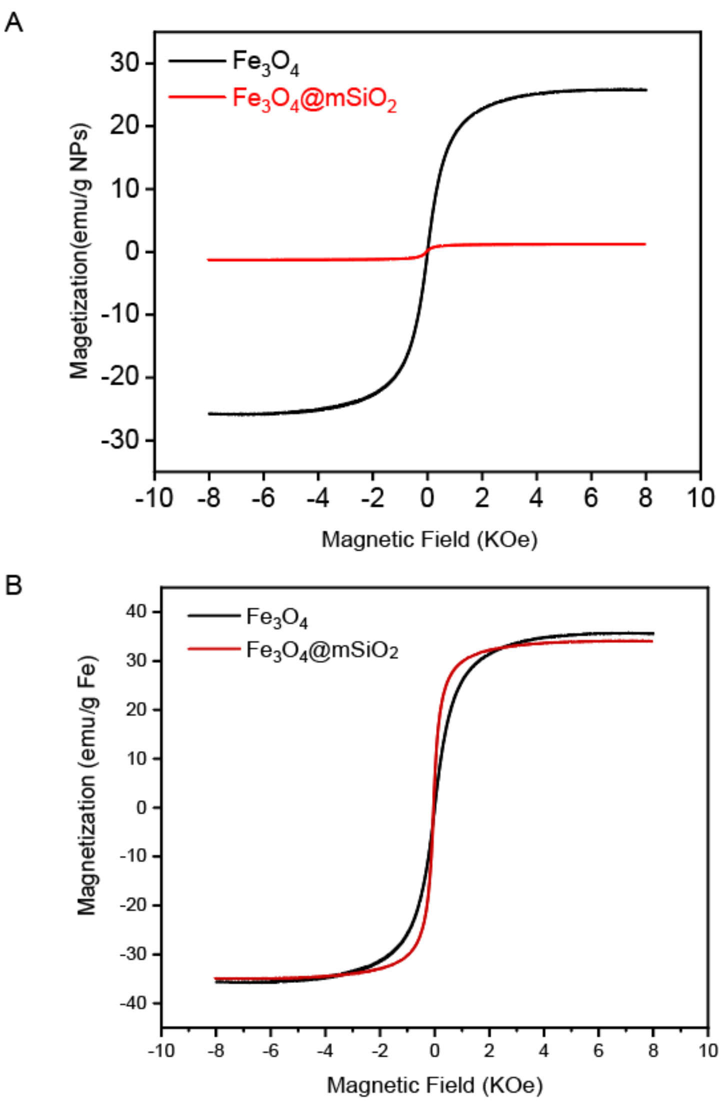

Figure S7. The saturation magnetization curve of Fe_3O_4 and Fe_3O_4@$mSiO_2$. The vertical coordinates are respectively the magnetization (emu/g NPs) in S7A and the magnetization (emu/g Fe) in S7B. The content of Fe element in Fe3O4 and Fe3O4@mSiO2 is 72.4% and 3.6%, respectively.

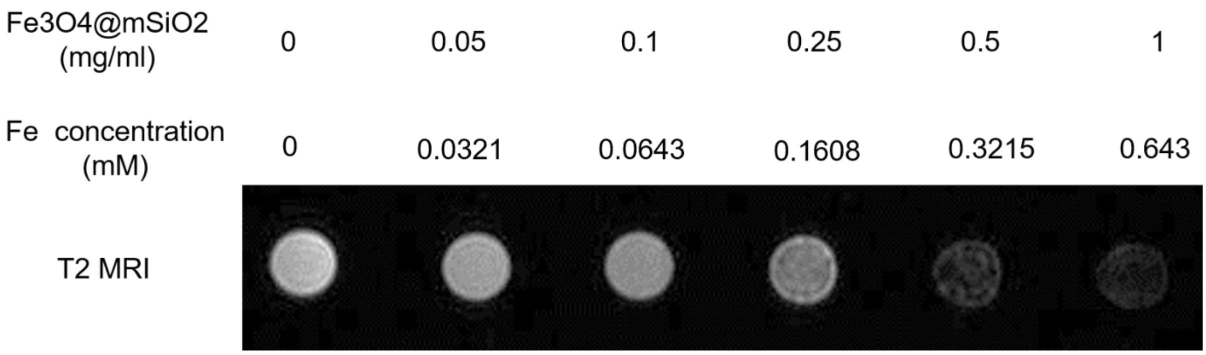

Figure S8. *In vitro* T2-weighted MRI of MNPs at different Fe concentrations (mM).

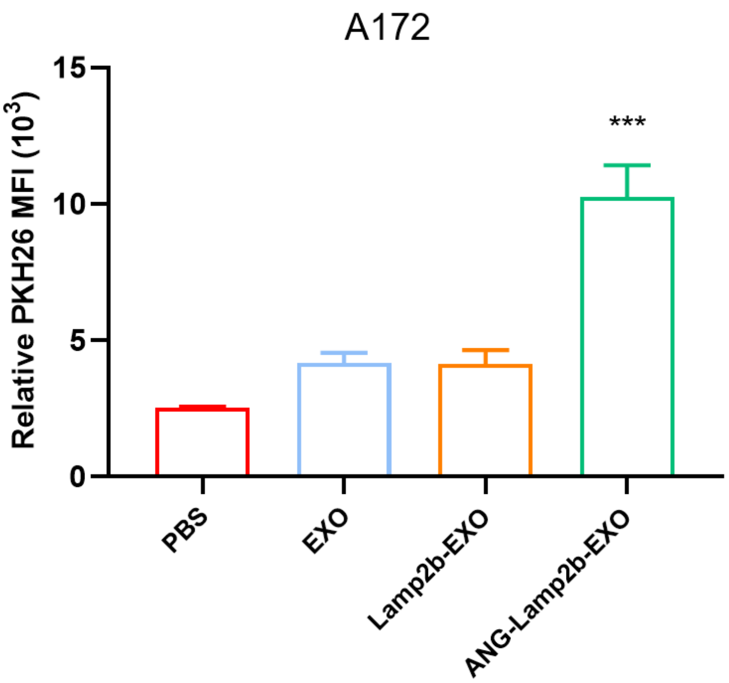

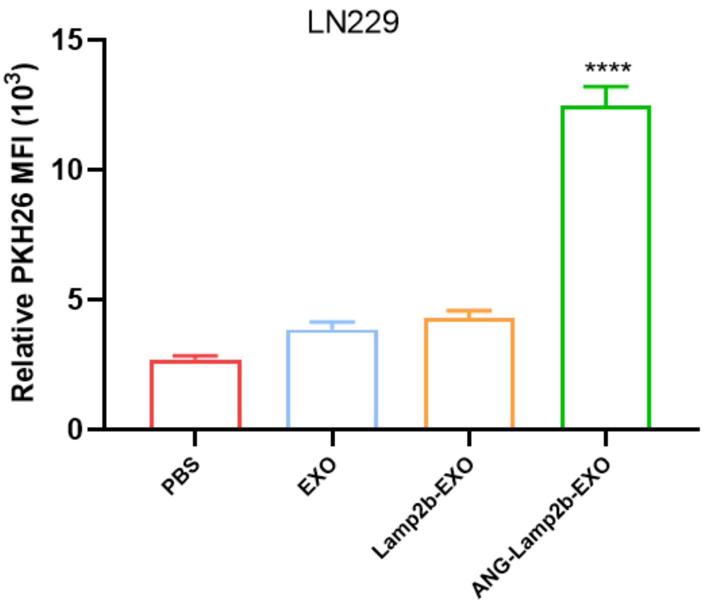

Figure S9. Quantification of mean fluorescence intensity (MFI) values in PKH26-labeled exosomes on A172 and LN229 cells (n=3 independent samples). Data are presented as mean ± s.d., and the difference between averages is significant by t test (***P < 0.001, ****P < 0.0001, compared with PBS group).

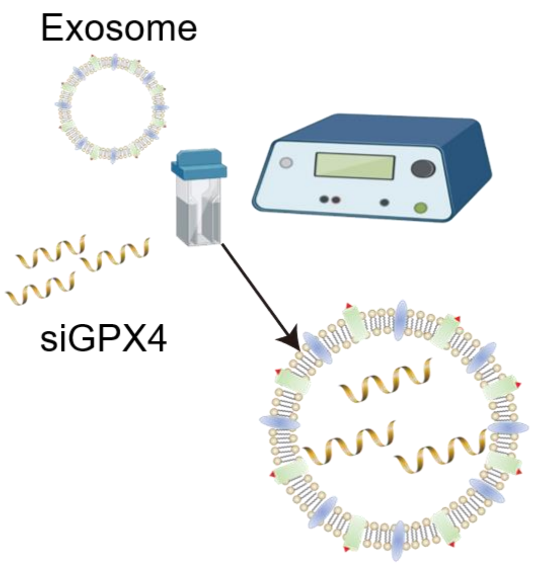

Figure S10. Schematic diagram of the electroporation of siGPX4 into exosomes.

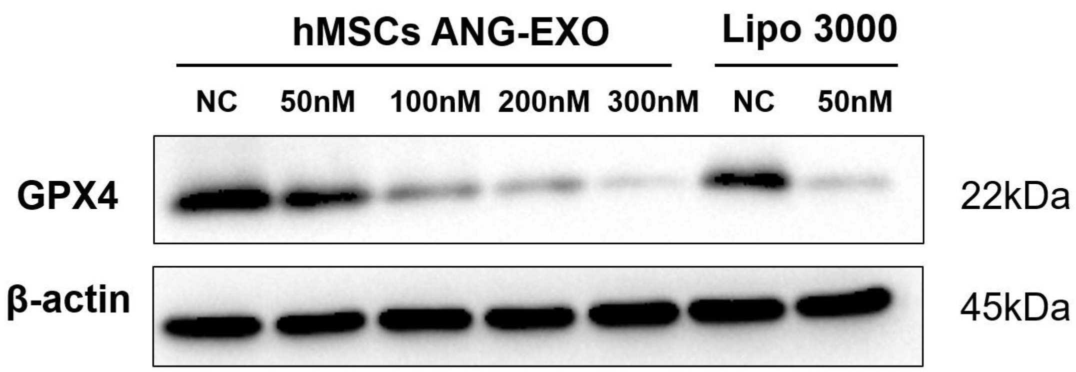

Figure S11. GPX4 Gene-silencing efficiency of hMSCs ANG-exosome determined via western blot.

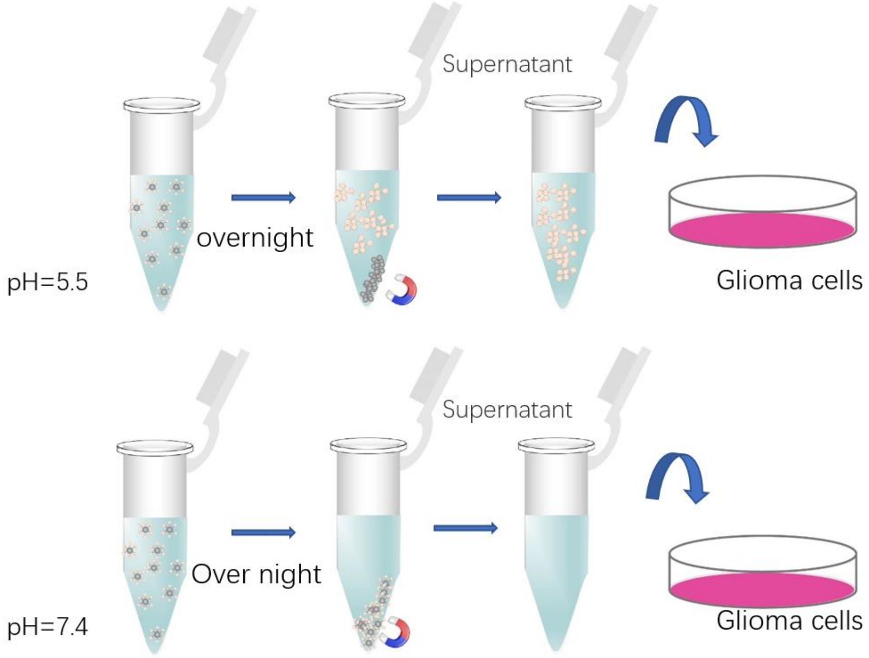

Figure S12. Schematic diagram of the *in vitro* guided test of the composite materials to verify the stability of the composites.

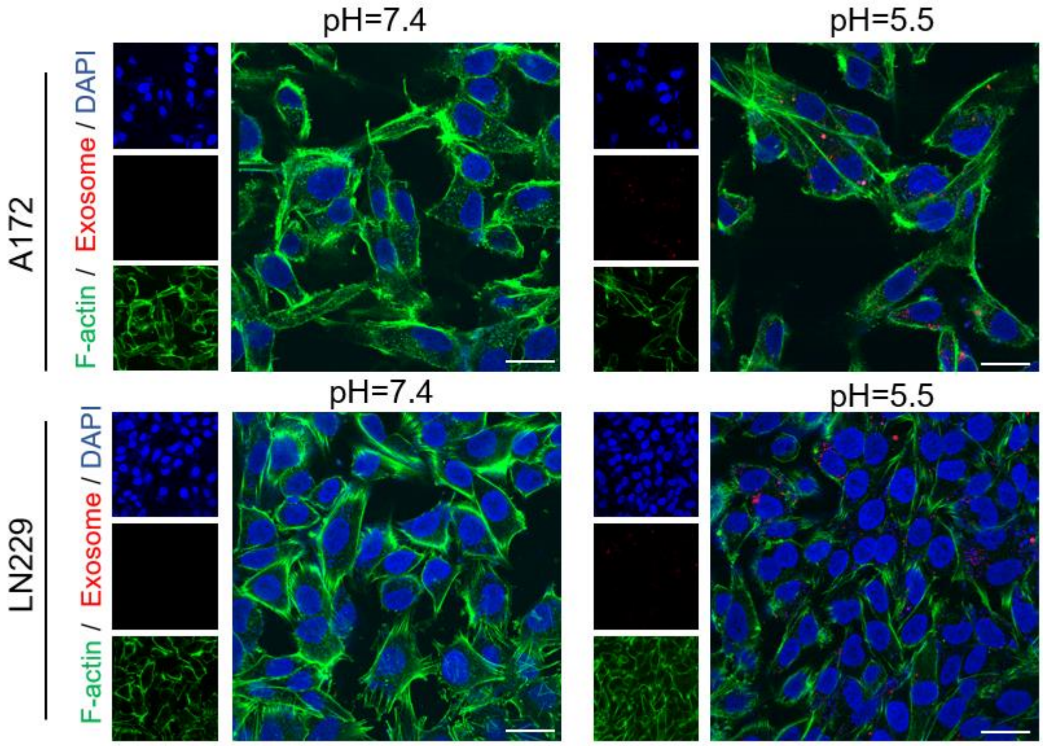

Figure S13. Confocal microscopy imaging of the composite material *in vitro* guided test. The GBM cells uptake of PKH26-labeled exosome release in acidic buffer (pH 5.5; right) but not in neutral buffer (pH 7.4; left). Scale bar, 25 µm.

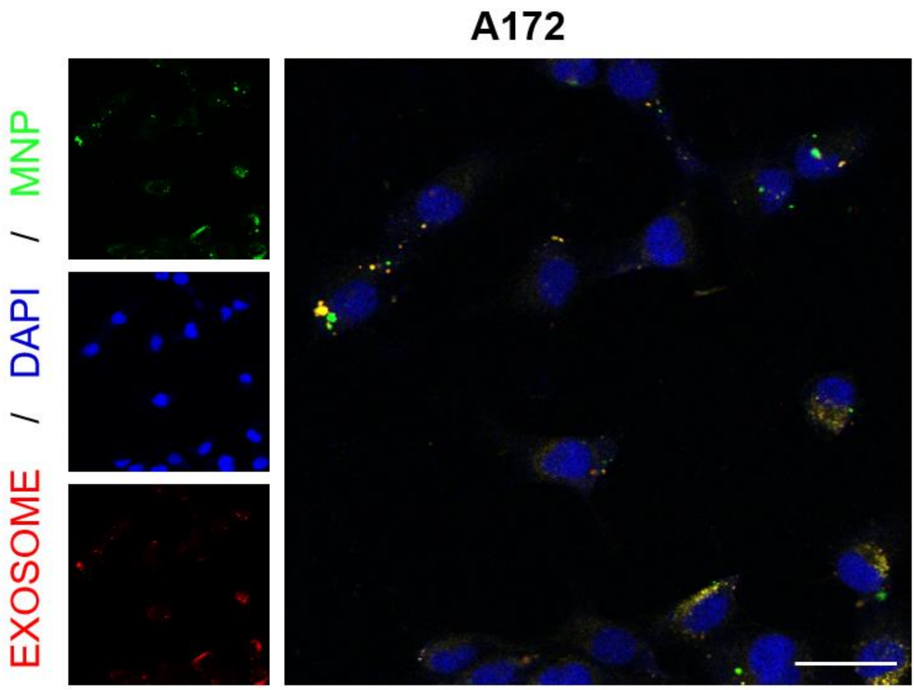

Figure S14. Cellular uptake of MNP@ANG-EXO after 6h incubation with A172 cells. Scale bar: 25 μm.

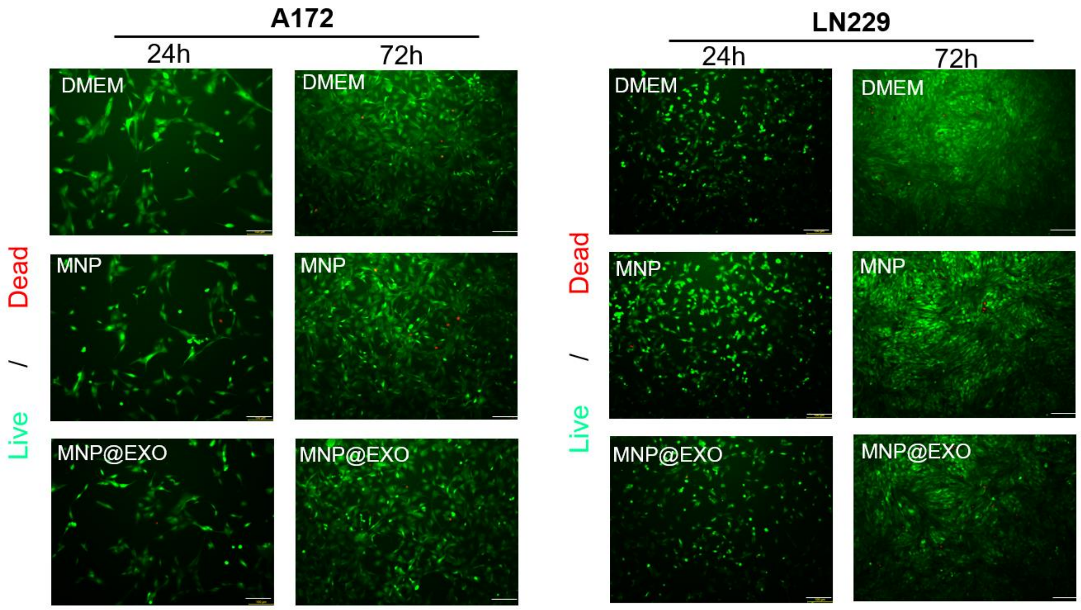

Figure S15. Representative live/dead staining images of the A172 and LN229 cells after culturing with culture medium, MNP and MNP@EXO for 24h and 72h. The live cells are stained green, and the dead cells are stained red. Scale bar: 100 μm.

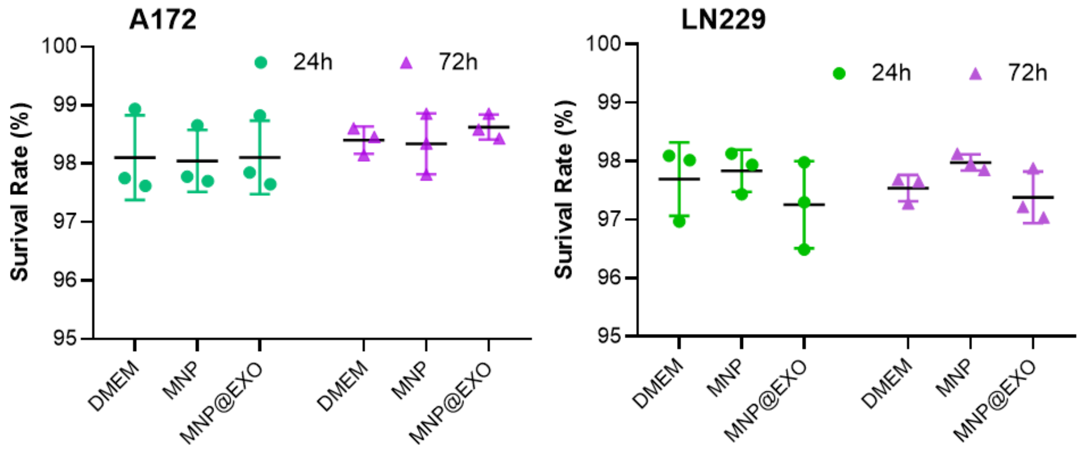

Figure S16. Quantification of Live/dead cells ration on confocal microscopy images. Data are presented as mean ± s.d. (n=3 independent samples).

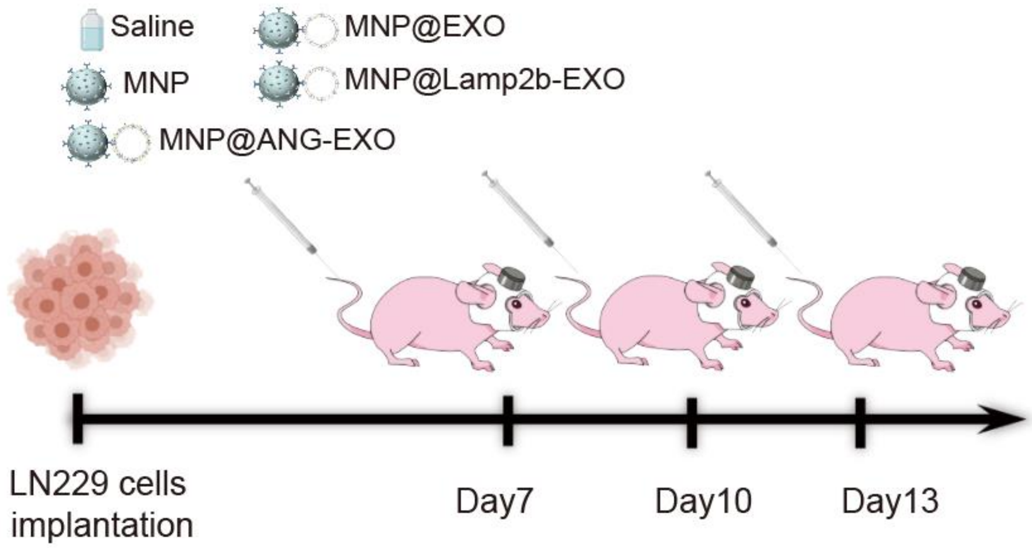

Figure S17. Schematic of the design of the animal experiments to examine the ability of MNP@ANG-EXO to cross the BBB and target the tumor.

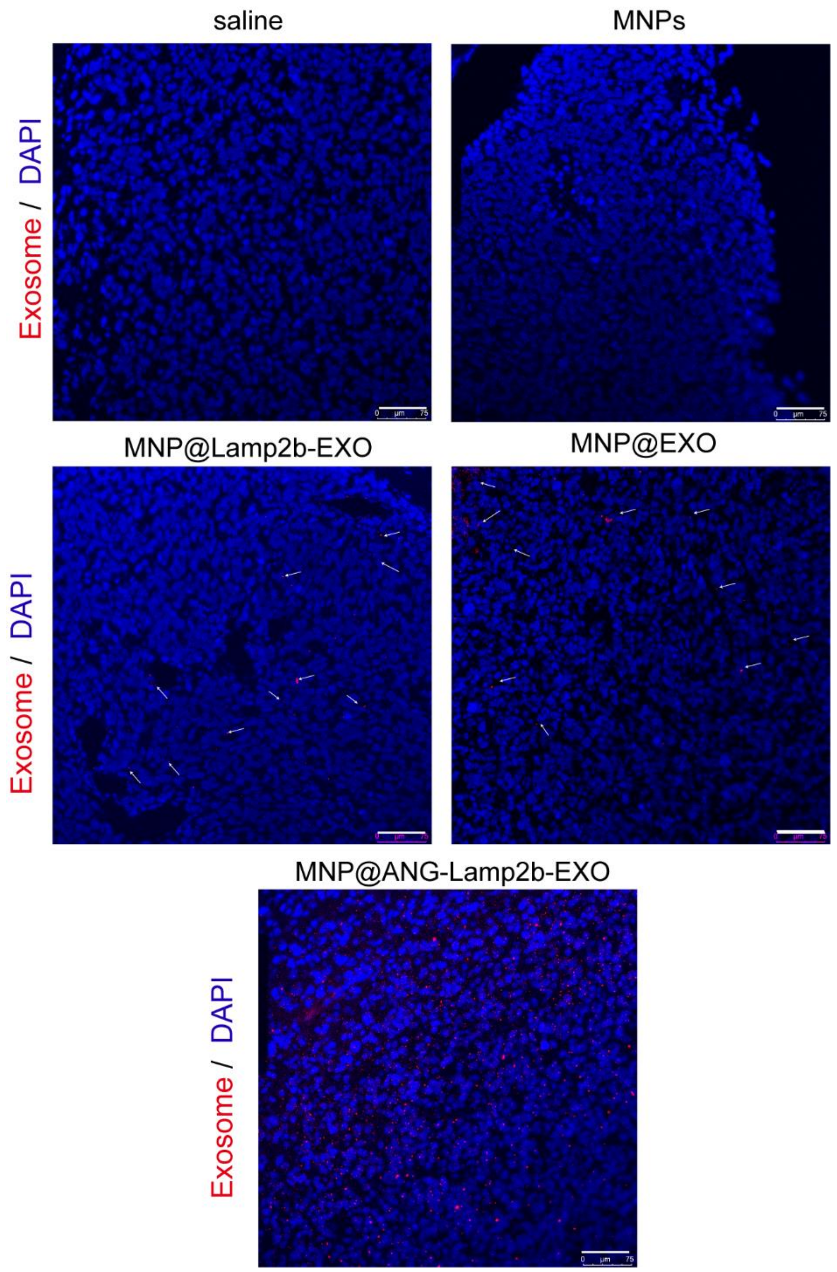

Figure S18. Confocal microscopy images showing PKH26-labeled exosomes in GBM tissue after injection of different materials. Scale bar, 75 μm

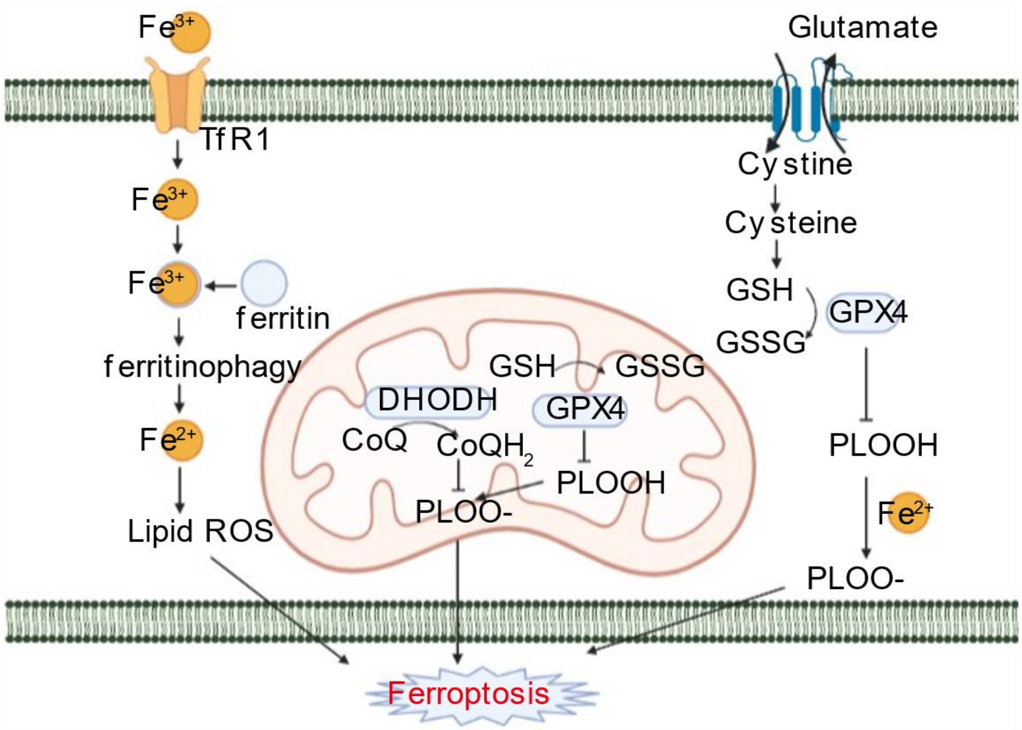

Figure S19. Schematic diagram of the ferroptosis pathway of DHODH and GPX4.

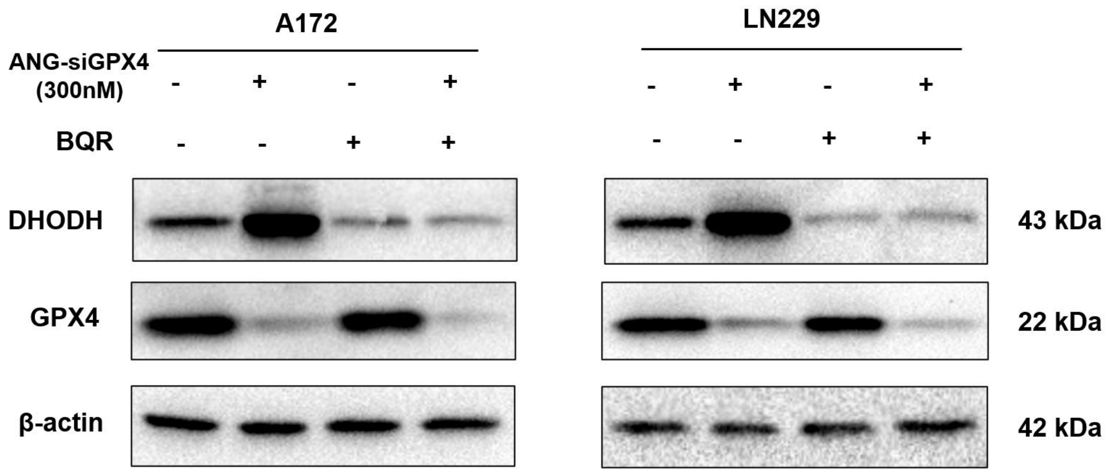

Figure S20. Western blot shows the protein expression of DHODH and GPX4 in A172 and LN229 cells after the addition of BQR and ANG-siGPX4 (300nM), respectively.

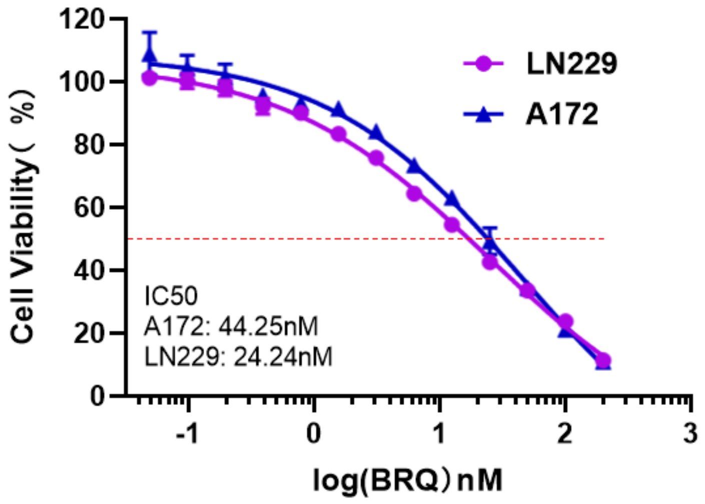

Figure S21. The IC50 of BQR in A172 and LN229 cells.

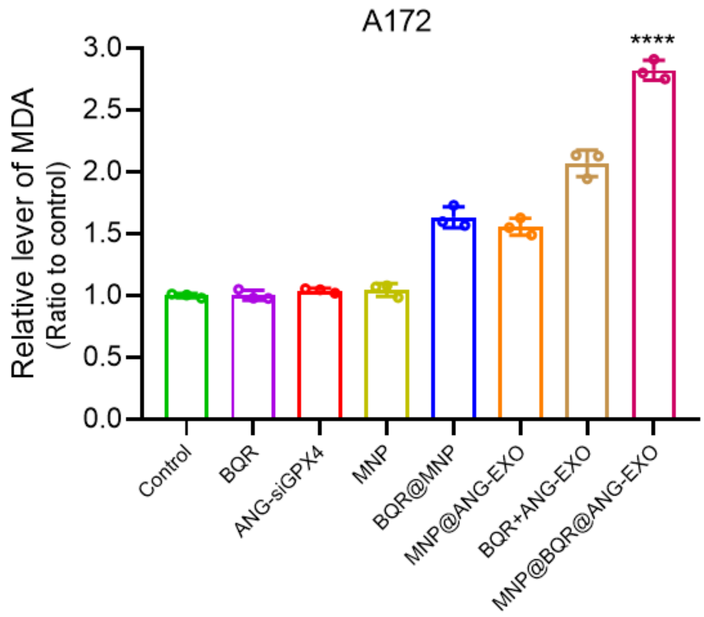

Figure S22. MDA level detected in A172 cells after co-culture with different nanoparticles, respectively. (n=3 and were normalized to the level in the control group; **** *P* <0.0001, compared with control group).

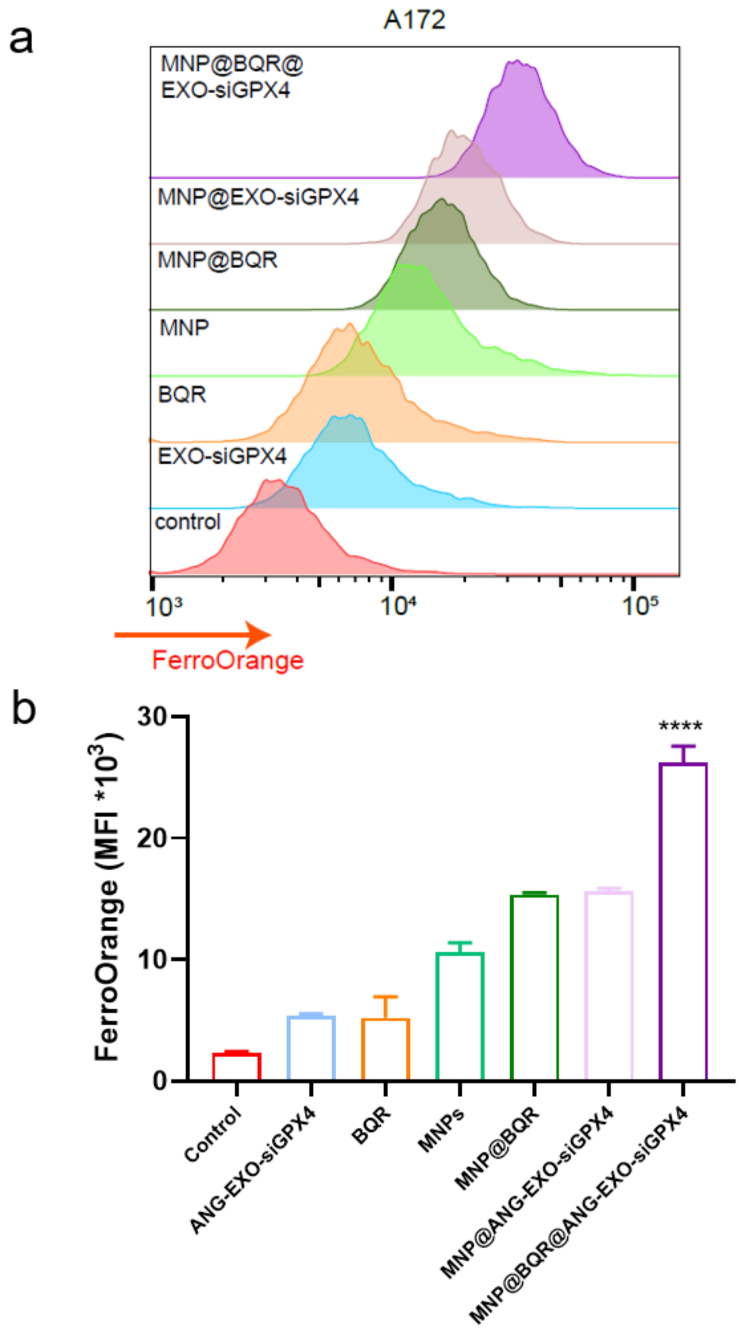

Figure S23. a) Flow Cytometry analysis for Fe^{2+}(FerroOrange staining) in A172 cells incubation with different nanoparticles, respectively. b) Quantification of mean fluorescence intensity (MFI) values in FerroOrange on A172 cells (n=3 independent samples and were normalized to the level in the control group). Data are presented as mean ± s.d., and the difference between averages is significant by t test (*****P* < 0.0001).

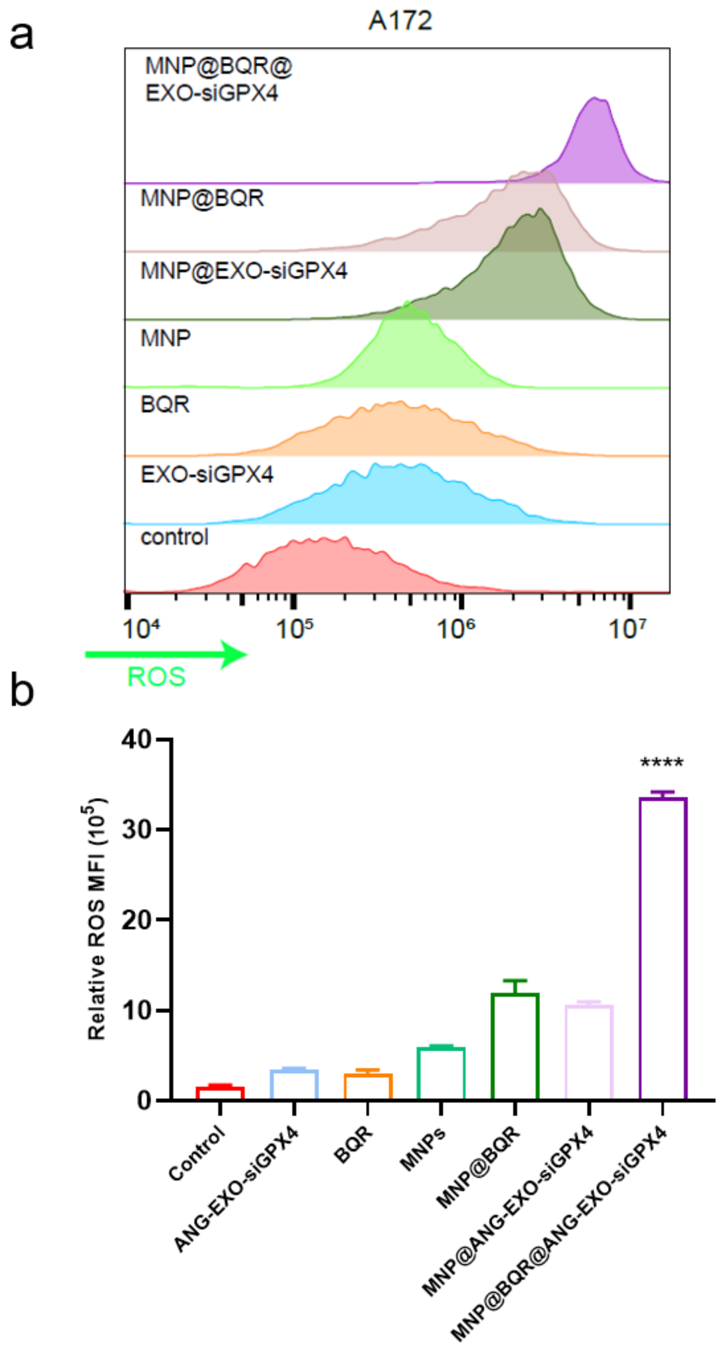

Figure S24. a) Flow Cytometry analysis for ROS (DCFH-DA staining) in A172 cells incubation with different nanoparticles, respectively. b) Quantification of mean fluorescence intensity (MFI) values in ROS on A172 cells (n=3 independent samples and were normalized to the level in the control group). Data are presented as mean ± s.d., and the difference between averages is significant by t test (*****P* < 0.0001).

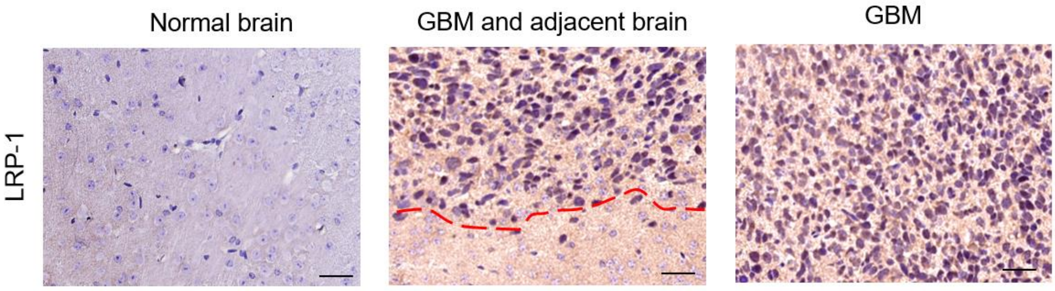

Figure S25. LRP-1 protein immunochemistry images of the normal brain, GBM and adjacent brain, GBM. Scale bar: 50 µm.

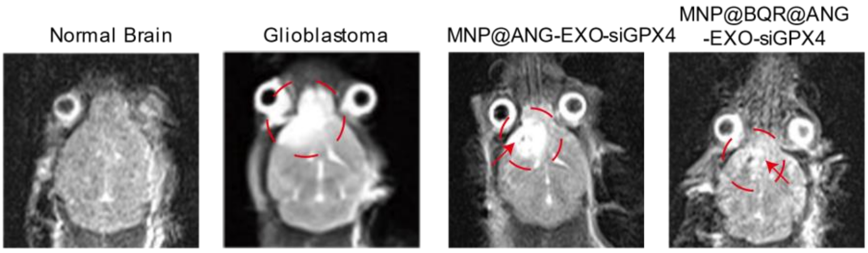

Figure S26. T2-weighted MRI of healthy mice and GBM mice after treatment. The red circle indicates the tumor, and the red arrows show the NPs.

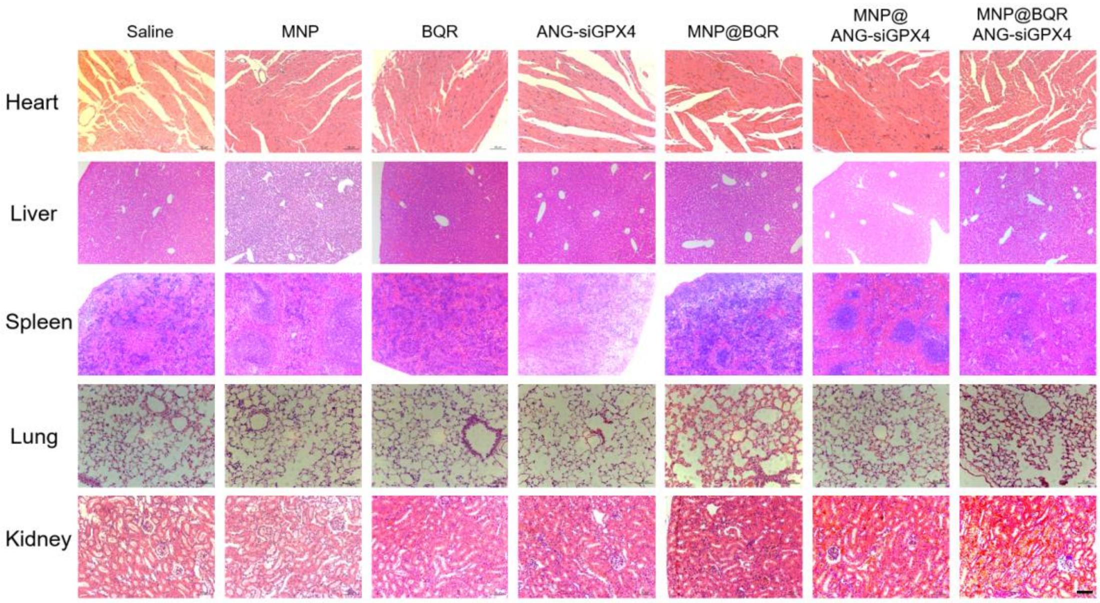

Figure S27. Representative images of H&E-stained sections from the heart, liver, kidney, spleen, and lung of tumor-bearing mice. Scale bar: 100 μm.

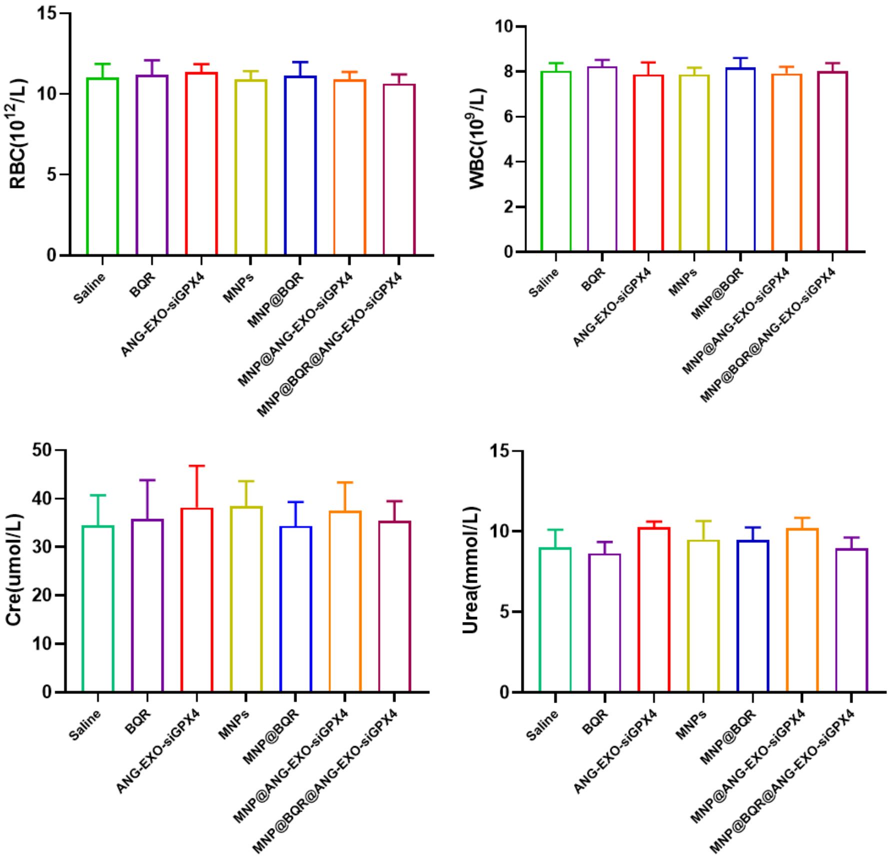

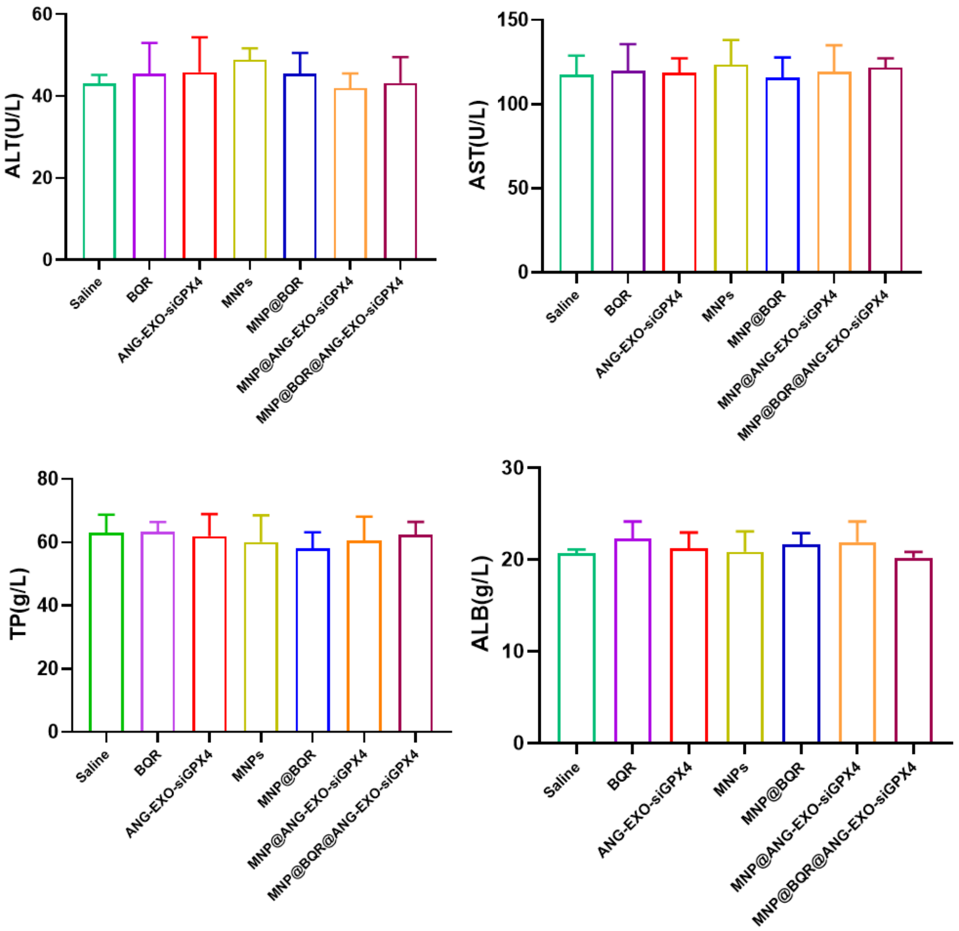

Figure S28. The toxicity of the nanomedicines evaluated by blood routine and blood biochemical index level.

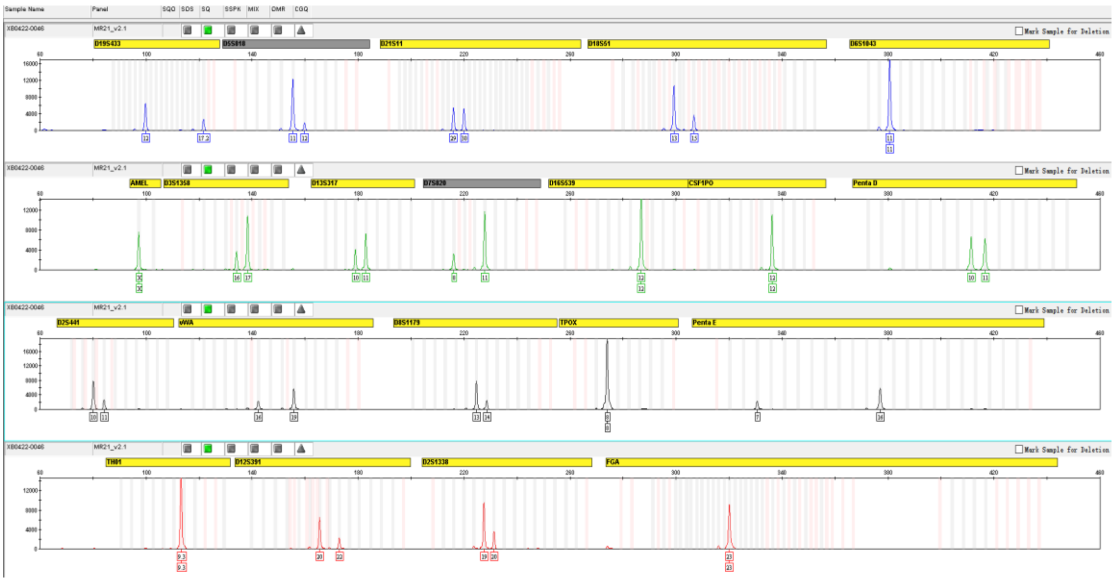

Figure S29. STR profiles of LN229 cell line.

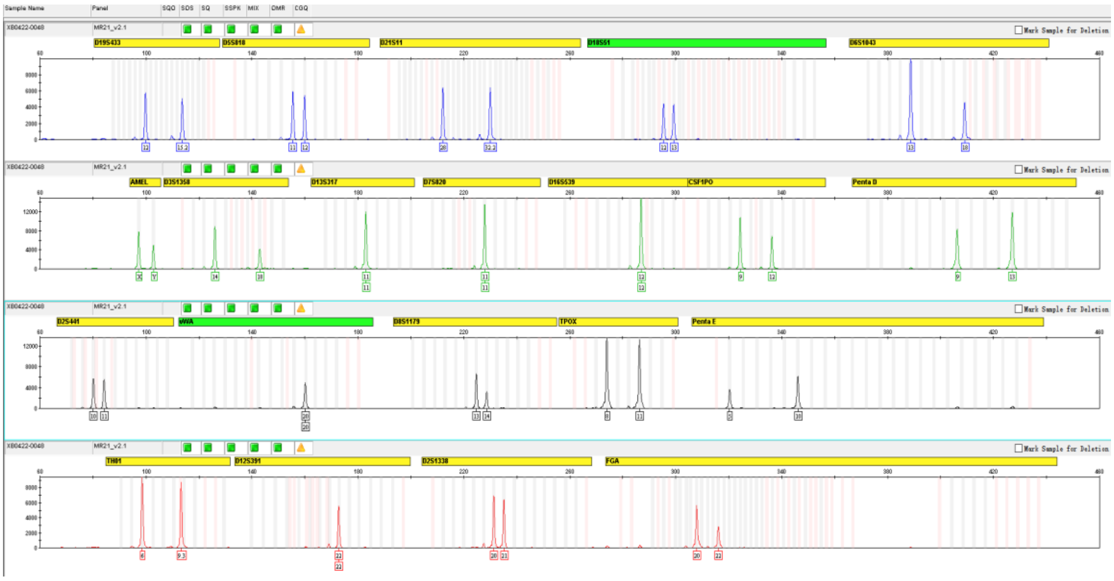

Figure S30. STR profiles of A172 cell line.
